# Quantum interference and heteroaromaticity of *para*- and *meta*-linked bridged biphenyl units in single molecular conductance measurements

**DOI:** 10.1038/s41598-017-01903-0

**Published:** 2017-05-11

**Authors:** Markus Gantenbein, Lin Wang, Alaa A. Al-jobory, Ali K. Ismael, Colin J. Lambert, Wenjing Hong, Martin R. Bryce

**Affiliations:** 1 Department of Chemistry, Durham University, Durham, DH1 3LE UK; 2 Department of Chemistry and Biochemistry, University of Bern, Freiestrasse 3, Bern, CH-3012 Switzerland; 3 Department of Physics, Lancaster University, Lancaster, LA1 4YB UK; 4 Department of Physics, College of Education for Pure Science, Anbar University, Anbar, Iraq; 5 Department of Physics, College of Education for Pure Science, Tikrit University, Tikrit, Iraq; 6 Collaborative Innovation Center of Chemistry for Energy Materials, Department Chemical and Biochemical Engineering, College of Chemistry and Chemical Engineering, Xiamen University, Xiamen, 361005 China; 7 Key Laboratory of Molecular Nanostructure and Nanotechnology, Institute of Chemistry Chinese Academy of Sciences, Beijing, 100190 China; 8 University of the Chinese Academy of Sciences, Beijing, China

## Abstract

Is there a correlation between the (hetero)aromaticity of the core of a molecule and its conductance in a single molecular junction? To address this question, which is of fundamental interest in molecular electronics, oligo(arylene-ethynylene) (OAE) molecular wires have been synthesized with core units comprising dibenzothiophene, carbazole, dibenzofuran and fluorene. The biphenyl core has been studied for comparison. Two isomeric series have been obtained with 4-ethynylpyridine units linked to the core either at *para-para* positions (*para* series **1–5**) or *meta-meta* positions (*meta* series **6–10**). A combined experimental and computational study, using mechanically controlled break junction measurements and density functional theory calculations, demonstrates consistently higher conductance in the *para* series compared to the *meta* series: this is in agreement with increased conjugation of the *π–*system in the *para* series. Within the *para* series conductance increases in the order of decreasing heteroaromaticity (dibenzothiophene < carbazole < dibenzofuran). However, the sequence is very different in the *meta* series, where dibenzothiophene ≈ dibenzofuran < carbazole. Excellent agreement between theoretical and experimental conductance values is obtained. Our study establishes that both quantum interference and heteroaromaticity in the molecular core units play important and inter-related roles in determining the conductance of single molecular junctions.

## Introduction

The measurement and understanding of charge transport in single molecules is of fundamental interest and is relevant to the proposed future applications of molecules in electronic devices^1–6^. Many studies have addressed correlations between molecular structure and transport properties of molecules wired into metal–molecule–metal nanoscale junctions^7, 8^. Several experimental approaches are well established for measuring transport through single (or a few) molecules, notably the mechanically controlled break junction (MCBJ)^9^ and scanning tunnelling microscopy-break junction (STM-BJ) techniques^10^. Combined experimental and theoretical studies^11^ have established that charge transport through molecular junctions is controlled by the intrinsic properties of the molecular backbone, the terminal anchoring group, and the metal leads. Key features are the molecular length, the molecular conformation, the gap between the highest occupied and the lowest unoccupied molecular orbitals (the HOMO-LUMO gap), the alignment of this gap to the Fermi level of the metal electrodes, and the coordination geometry at the metal-molecule contacts. Oligo(arylene-ethynylene) (OAE)-type molecular wires have been widely explored in single molecular junctions^12–16^. They are *π*–conjugated, rod-like molecules and their functional properties can be systematically tuned over a wide range of parameters by chemical synthesis^17^.

**Figure 1:**
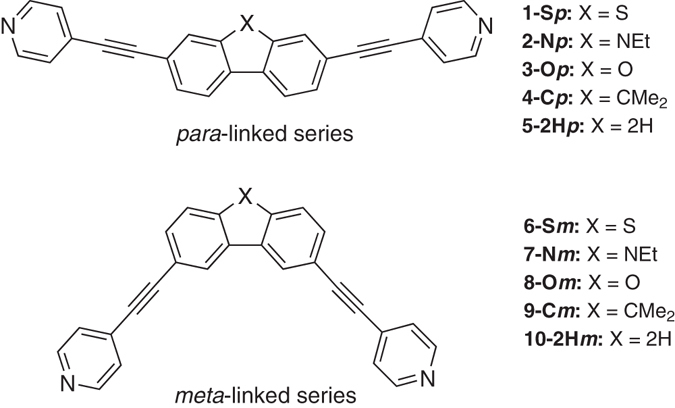
Structures of the molecules discussed in this work and their nomenclature. The structures represent the *para*-linked series **1–5** (top) and *meta*-linked series **6–10** (bottom).

In the present work we investigate a series of ten OAE molecules **1**–**10** whose structures are shown in Fig. 1. The molecular design combines three key structural features: (i) all of the molecules have terminal pyridyl anchoring units at both ends; (ii) each molecule has one of five different core units and (iii) there is either *para-para* or *meta-meta* conjugation through the core unit, providing two isomeric series. The dibenzothiophene (**1**, **6**), *N-*ethylcarbazole (**2**, **7**), dibenzofuran (**3**, **8**) and 9,9-dimethylfluorene cores (**4**, **9**) are rigid and planar. Heteroaromaticity, i.e., the resonance energy, will decrease in the sequence dibenzothiophene > carbazole > dibenzofuran, reflecting the extent of delocalization of a lone pair from the heteroatom into the *π–*system of the central ring (S > N > O)^18^. Fluorene, with no heteroatom and a bridging sp^3^ carbon atom instead, has a non-aromatic central ring. In contrast to the other molecules in Fig. 1, biphenyl derivatives **5** and **10** possess a flexible and twisted core. It is well known that increasing the torsion angle within a biphenyl unit leads to reduced single-molecule conductance^19–23^, therefore, **5** and **10** are studied here as model compounds.

**Figure 2:**
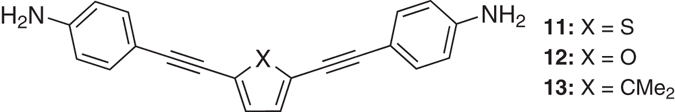
Molecules studied in ref. 24. Amine-terminated molecules **11–13** measured by STM-BJ.

We are aware of only two related reports on the effect of heteroaromaticity on single-molecule conductance. Venkataraman, Breslow and co-workers studied three amine-terminated molecules comprising thiophene, furan and dimethylcyclopentadiene cores (**11–13**, Fig. 2). Based on STM–BJ measurements the authors concluded that aromaticity in the core leads to a decrease in the single-molecule conductance, i.e. the non-aromatic cyclopentadiene derivative **13** has the highest conductance, while the most aromatic thiophene derivative **11** has the lowest conductance^24^. This work did not consider the linkage of the anchor units to different positions on the core.

A second study concerns multiple pathways through a molecular wire based on fluorene-like molecules^25^. Several studies have established that *para* (conjugated) connectivity through a core unit results in enhanced conductance compared to the isomeric *meta* (reduced conjugation) connectivity. This is ascribed to quantum interference and has been observed experimentally and theoretically in aromatic rings such as benzene^26–29^, naphthalene^14^, anthracene^14^, pyrene^30^ and anthanthrene^31^.

The motivation for the present work is to study for the first time the combined effects of two important molecular parameters on the single-molecule conductance of molecular wires: (i) heteroaromaticity in the core of the wire, and (ii) *para* versus *meta* conjugation through the core unit.

## Results

### Synthesis

For the synthesis of the *para*-linked **1–5** and *meta*-linked **6–10** molecules (Scheme 1) a tandem two-pot reaction sequence was followed. To enable the Sonogashira cross coupling of the corresponding aryldibromide central units (structures **14–23** ^32–39^ in SI) at elevated temperatures, desilylation of 4-((trimethylsilyl)ethynyl)pyridine^40^ with TBAF (1 M in THF) was performed in 1,4-dioxane (room temperature for 30 min). Since the light-sensitive 4-ethynylpyridine is not stable in air, the mixture was directly subjected to the aryldibromide in the presence of PdCl_2_(PhCN)_2_, CuI, *t*-Bu_3_P, and (*i*-Pr)_2_NH, to give the target structures **1–10** in very good yields (Supplementary Note 1). All the compounds were characterized by ^1^H and ^13^C NMR spectroscopy, mass spectrometry and elemental analysis. In addition, to assess the extent of conjugation within compounds **1–10** their UV–Vis absorption spectra were measured in a dilute and aerated dichloromethane solution at room temperature (Supplementary Figure 1). The optical HOMO-LUMO gaps (*E*_g_) were calculated from the onset of the absorption and are listed in Supplementary Table 1. Compounds **1–5**, with the anchor groups attached in the *para* positions, show a smaller HOMO–LUMO gap compared to the *meta* isomers **6–10**. This is consistent with reduced conjugation in the *meta* series.

### Single-molecule Conductance Measurements

**Figure 3:**
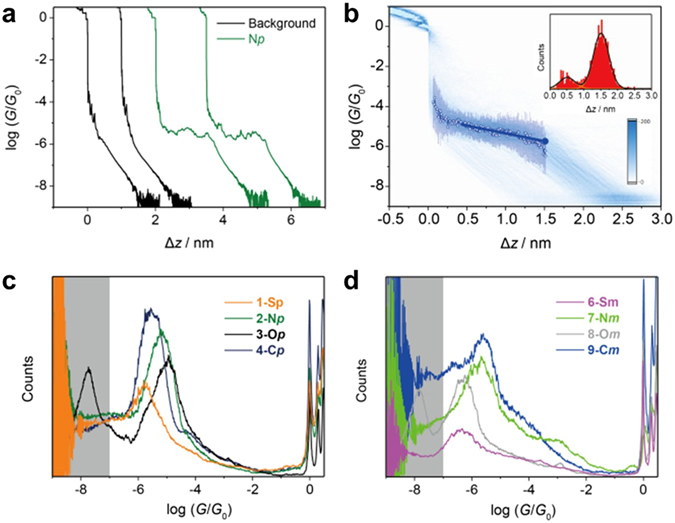
Single-molecule conductance results from MCBJ experiments. (**a**) Typical individual conductance–distance traces (horizontally offset for clarity) of **N** ***p*** (green) and pure tunneling traces (black). (**b**) All-data-point 2D conductance versus relative distance (Δ*z*) of **N** ***p***. In 2D histogram, statistically averaged conductance–distance traces (hollow circles) with variations indicated by the standard deviations (bars) are shown, along with the linear fit (line). The solid circle represents the last data point in the linear fit before junction rupture. Inset: Stretching distance distribution obtained between 10^−0.30^ *G*_0_ and 10^−6.25^ *G*_0_. (**c**) and (**d**) All-data-point 1D conductance histograms constructed from more than 1,000 MCBJ traces of molecules with anchoring groups on (**c**) *para-para* and (**d**) *meta–meta* position. The gray area represents the detection limit of the MCBJ set up at 10^−7^ *G*_0_.

Single-molecule conductance measurements of **1–10** in molecular junctions were performed using a home-built mechanically controllable break junction (MCBJ) setup at a bias *V*_*bias*_ = 0.1 V. Figure 3a shows typical individual conductance *G* (in units of quantum point conductance *G*_0_ = 2e^2^/h) versus distance (Δ*z*) stretching traces in the measurement of **N** ***p***. The conductance in the molecule-free traces (black line) reveals exponential decrease characteristics upon the stretching process. When molecule **N** ***p*** is present a pronounced conductance plateau around 10^−5^ *G*_0_ could be detected (green line) after the Au–Au contact breaks, which is assigned to the gold–molecule–gold junction. Since the break junction method can create a large number of molecular junctions with different molecule–electrode contact geometries, more than 1000 curves were recorded for statistical analysis to determine the most probable conductance of the molecular junctions. We further introduced a relative distance (Δ*z*) and defined Δ*z* = 0 at 0.5 *G*_0_ to align all the traces. This procedure leads to an accurate alignment of the conductance–distance traces because of the sharp drop in conductance at *G* < *G*_0_. The electrode separation *z*_exp_ is then estimated by *z*_exp_ = Δ*z* + Δ*z*_*corr*_, where Δ*z*_*corr*_ = 0.5 ± 0.1 nm corresponds to the “snap-back” nanogap which forms immediately upon breaking of the gold-gold atomic contact^41^. The all–data two–dimensional (2D) histogram (Fig. 3b) exhibits features of gold–gold contacts around *G* ≥ 1 *G*_0_, followed by another well-defined conductance scatter group in the range of 10^−4^ *G*_0_~10^−6^ *G*_0_ which is attributed to the formation of single–molecule junctions. Figure 3c–d demonstrate the comparison between molecules with different bridging units and anchoring positions. For the compounds **1–3** with the *para–para* connectivity, the conductance clearly increases in the sequence **S** ***p*** < **N** ***p*** < **O** ***p***. However, for the isomers **6–8** where the anchoring groups are attached at *meta–meta* positions, the conductance reveals a different trend, **S** ***m*** ≈ **O** ***m*** < **N** ***m***. Control experiments using analogues with a carbon bridge (**4-C** ***p*** and **9-C** ***m***) and without any bridging atom (**5-2** **H** ***p*** and **10-2** **H** ***m***) were also conducted. The conductance of **2** **H** ***m*** could not be measured within the detection range of our setup. This can be explained by the *meta* coupling combined with a non-planar biphenyl core giving a conductance value below the direct tunneling conductance^29^.

As the junction configuration is known to have a significant effect on the single–molecule conductance, we further explored the master curves composed of the fitted conductance with standard variation at each cross-sectional distance point^42^. After a linear fitting, the **N** ***p*** junction conductance with a fully stretched molecular conformation before the junction rupture can be deduced as 10^−5.74±0.17^ *G*_0_, which should be closer to the theoretical predicted configurations.

It is found that there is some difference in the conductance comparison among different molecules: **O** ***m*** shows lower conductance than **S** ***m*** for the conductance of the fully-stretched configurations, while **O** ***m*** shows a slightly higher conductance for the most probable conductance extracted from conductance histogram.

The key results of the MCBJ measurements are summarized in Table 1 and the corresponding original results are presented in the Supporting Information (Supplementary Figures 24–33). No multiple features were observed in the experiments, including **3-O** ***p*** and **8-O** ***m***. The lower peaks covered by the grey area below 10^−7^ *G*_0_ is the noise level of the MCBJ experiments (Fig. 3(c,d)). All the curves were used for the statistical analysis without any data selection. Junction formation probability (JFP) is the proportion of molecular stretching traces with a pronounced plateau relative to the total number of traces (Table 1). It is judged by area ratio of the peak in the plateau length distribution. Direct tunneling traces have no plateau and decay faster to the noise level, corresponding to the smaller stretching peak alongside the molecular peak in the plateau length histogram. (Supplementary Figures 24–33).

**Table 1:** Most Probable Conductance Values as Obtained from MCBJ Experiments and Computations.

Compound	MCBJ (log(*G*/*G*_0_))		DFT (log(*G*/*G*_0_))		*G*_*F para*_ * − G*_*F meta*_ (log(*G*/*G*_0_))^*c*^		JFP (%)^*d*^	Electrode separation *z*_exp_ = Δ*z* + Δ*z*_corr_ (nm)^*e*^	Theoretical length (nm)
	*G*_*M*_^*a*^	*G*_*F*_^*b*^	*G*_*M*_	*G*_*F*_	MCBJ	DFT			
**1-S** ***p***	−5.79 ± 0.30	−5.93 ± 0.16	−5.50	−5.74	0.72	0.66	69	2.04 ± 0.45	2.04
**2-N** ***p***	−5.22 ± 0.40	−5.74 ± 0.17	−5.15	−5.36	0.67	0.70	83	2.01 ± 0.23	2.02
**3-O** ***p***	−5.04 ± 0.47	−5.47 ± 0.22	−5.10	−5.33	1.37	1.19	99	1.92 ± 0.26	2.03
**4-C** ***p***	−5.55 ± 0.44	−5.81 ± 0.12	−5.44	−5.66	0.09	0.68	70	1.84 ± 0.22	2.03
**5-2H** ***p***	−5.40 ± 0.36	−5.68 ± 0.11	−5.30	−5.55	>1.32	2.34	83	2.11 ± 0.23	2.08
**6-S** ***m***	−6.34 ± 0.47	−6.65 ± 0.35	−6.34	−6.40			100	1.64 ± 0.29	1.51
**7-N** ***m***	−5.76 ± 0.48	−6.41 ± 0.25	−6.06	−6.06			71	1.66 ± 0.14	1.56
**8-O** ***m***	−6.32 ± 0.38	−6.84 ± 0.24	−6.40	−6.52			100	1.65 ± 0.19	1.55
**9-C** ***m***	−5.66 ± 0.82	−5.90 ± 0.39	−5.74	−6.34			100	1.46 ± 0.29	1.51
**10-2H** ***m***	<−7	<−7	−7.38	−7.89			—	—	1.41

^*a*^Conductance fitted by Gaussian function in the 1D histogram. ^*b*^Molecular conductance through fully stretched junction obtained by linear fitting of the master curve. ^*c*^Fully-stretched conductance difference between *para*- and *meta*-linked molecule for each bridging unit. ^*d*^Junction formation probability judged by area ratio of the peak in the plateau length distribution. ^*e*^Most-probable electrode separation at the end of conductance plateau. Error bars are based on the Gaussian fitting of conductance all-data-point 1D histogram and plateau displacement distribution.

Several interesting conclusions can be drawn from the comparative conductance values of these molecules. First, in all cases, molecules with *para* connectivity **1–5** present larger conductance values than their *meta* isomers, regardless of the bridging unit. This can be attributed to the partial de Broglie waves traversing in different paths through the core being in phase in the *para* isomers, giving rise to a constructive quantum interference (QI) effect. On the contrary, in the *meta*-anchored isomers the waves are out of phase leading to destructive quantum interference. The conductance relationship of the *para* and *meta* molecules is consistent with that of molecules with a central single benzene ring^27–29, 43^, indicating the quantum interference effect can still operate in polycyclic compounds. Secondly, the structure of the central core plays an important role in the conductance of QI molecules. It is noted that the largest difference between the *para*– and *meta*–anchored molecules (Δ*G*) is for the dibenzofuran pair **3-O** ***p*** and **8-O** ***m***, 1.37 log(*G*/*G*_0_). However, as we reported previously, the differences between *para* and *meta* linked molecules are nearly 1.50 log(*G*/*G*_0_) in benzene-cored analogs^29^. The lower experimental differences in the present study demonstrate that the quantum interference effect has not been amplified, and is even slightly reduced, by bridging the two benzene rings with a five-membered ring. Additionally, differences of the conductance in fully-stretched conformations between *para*– and *meta*–anchored molecules follow the increasing order of Δ*G* **C** < Δ*G* **N** < Δ*G* **S** < Δ*G* **O**, illustrating that the heteroatom can also contribute to the expression of the quantum interference. The electrode separations (*z*_exp_ in Table 1) are in good agreement with the theoretical molecular lengths. This indicates that in the fully stretched configuration, the molecular junctions are primarily linked by the gold–nitrogen bonds.

Moreover, there is no distinct correlation between the plateau length (or JFP) and the nature of the bridging atoms (S, N, O or C), demonstrating that these atoms have no significant influence on the conformation of the molecular junction. Furthermore, we did not observe any additional conductance group during the experiments for the ten molecules. We attributed this fact to three reasons. Firstly, pyridyl-terminated compounds have been reported to show well-defined peaks in the conductance histograms resulting from the high directionality of the donor-acceptor binding between N lone pair and Au^15, 44, 45^. Secondly, the alkyl groups connected to the bridged atom (N and C) sterically hinder the interaction between the electrode and the core of the molecule as well as restricting any *π*-*π* interaction of two molecules. Thirdly, molecules with similar core structures have been reported^19, 22, 23^ to exhibit only one conductance statistical peak, suggesting that the junction formed by the core of the molecule is not robust enough during the elongation process. In the control experiments with **5-2** **H** ***p*** and **10-2** **H** ***m***, however, we observed such an obvious difference that the conductance of **5-2** **H** ***p*** is higher than that of **10-2** **H** ***m*** by almost two orders of magnitude.

### Theory and Simulations

**Figure 4:**
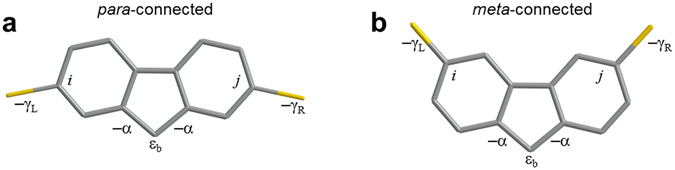
Tight-binding (i.e. Hückel) models of *para*-(**a**) and *meta*-connected (**b**) molecules. Within the core of each, all site energies are zero except the pendant site energy *ɛ*_b_ and all nearest neighbour bonds are equal to −1, except for those denoted as *α*. The weaker couplings between the molecule and left leads are −*γ*_L_ = −0.08 at atom number i and right leads −*γ*_R_ = −0.08 at atom number *j*.

To understand the effect of pendant groups on quantum interference in the molecules of Fig. 1, we first consider their two tight-binding representations shown in Fig. 4, connected to 1-dimensional external leads.

**Figure 5:**
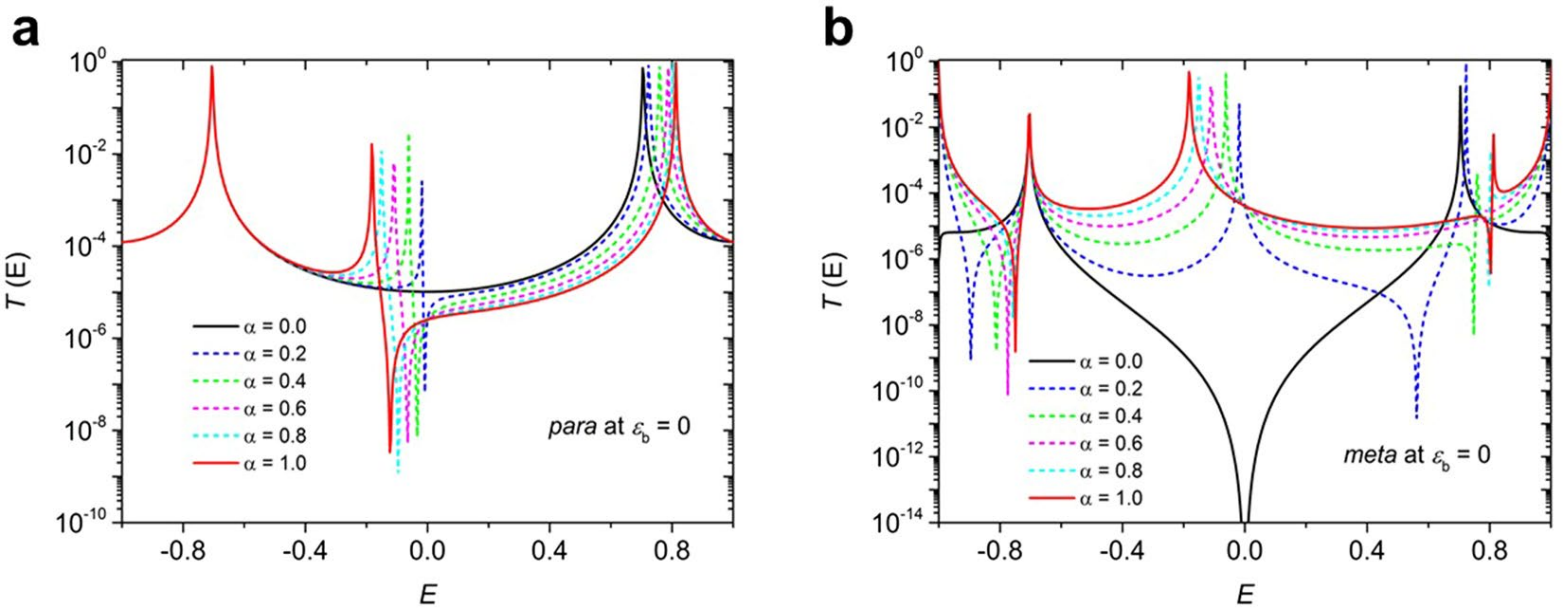
*T* (E) vs. *E* for different values of *α* at *ε*_b_ = 0. Transmission coefficients *T* (E) for *para*-connection (**a**) and *meta*-connection (**b**), when *ε*_b_ = 0.

**Figure 6:**
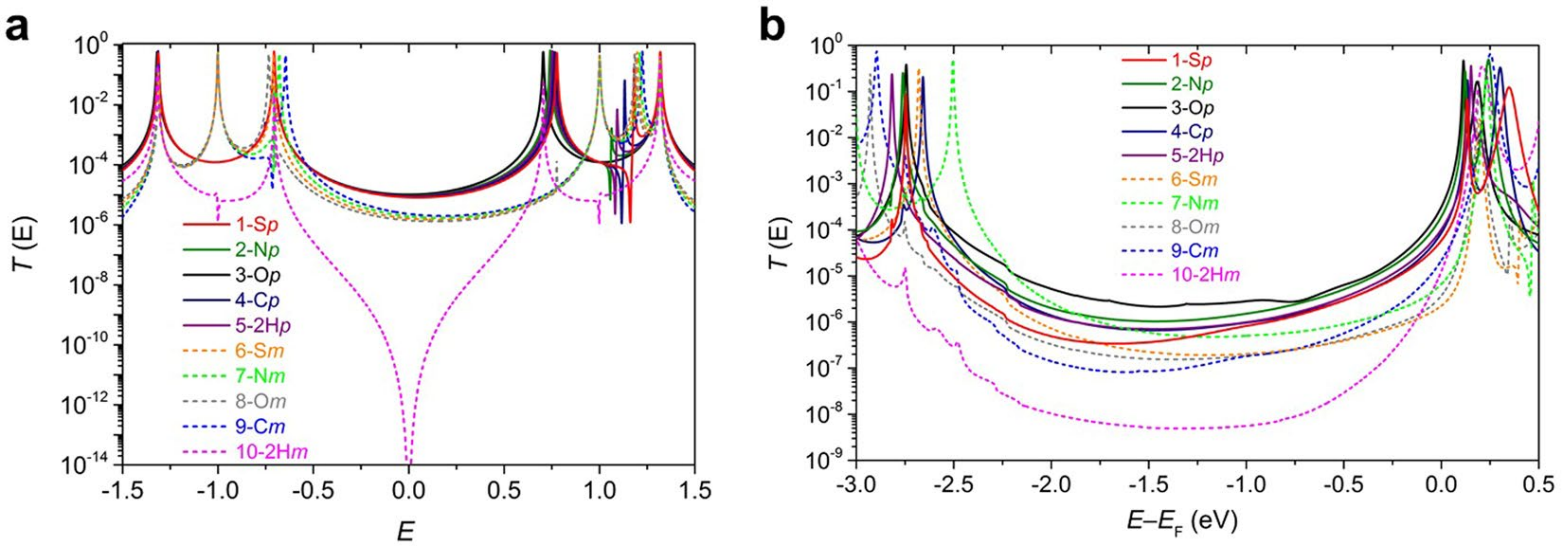
Transmission coefficients. Results for the tight-binding (Hückel-model) transmission coefficients (**a**) obtained using the parameters in Supplementary Table 5. The transmission coefficients (**b**) obtained for electrodes with adatoms (see Fig. 7b) using density functional theory combined with Gollum.

The tight binding model is introduced to illustrate the underlying trends in the transmission function and to allow us to obtain an analytic formula. Figure 5 shows results for various values of alpha, to reveal the evolution of the transmission curves with increasing coupling to the pendant groups. In Fig. 6a, to use the simplest possible description, the same value of alpha = 1 is used for all molecules (for more information see Supplementary Table 5). When *α* = 0, the pendant orbital is decoupled from the central core. Since the latter is a bipartite lattice, in the *meta* case, destructive interference should occur at the centre of the HOMO–LUMO gap (i.e. *E* = 0)^11, 30, 31^. The black curves in Fig. 5 show the resulting transmission coefficients *T* (E), when *ε*_b_ = 0. The other curves in Fig. 5 show how the transmission coefficient evolves as the coupling *α* to the pendant orbital is increased from zero (black curves) to unity (red curves).

The above results show that in the absence of pendant groups (i.e. when *α* = 0) the *meta* case shows a sharp transmission dip due to destructive interference at the gap centre, which is absent in the *para* case. In the presence of pendant groups (i.e. when *α* is non-zero) this destructive interference is alleviated in the *meta* case. In the *para* case, the non-zero coupling to the pendant group introduces a new conductance pathway, which can cause destructive interference within the gap, signaled by the Fano lineshape just below *E* = 0. Further examples of this evolution for different choices of *ε*_b_ are presented in the SI. For the values of *ε*_b_ shown in Supplementary Table 5, Fig. 6a, shows the resulting tight-binding transmission coefficients. Clearly the tight-binding model captures the qualitative features of the full density-functional calculation of transmission curves shown in Fig. 6b. In particular the tight-binding result for **2H** ***m***, which does not possess a pendant orbital, shows a pronounced transmission dip near *E* = 0, which is reflected in the low transmission coefficient predicted by DFT. (In the latter case, the presence of non-*pi* orbitals provides a parallel conductance path, which prevents the transmission coefficient completely vanishing.)

The electronic interference structure calculations leading to transmission curves (Fig. 6b) were performed using the DFT code SIESTA^46^. The optimum geometry of the isolated molecules was obtained by relaxing the molecules until all forces on the atoms were <0.05 V/Å. The SIESTA calculations employed a double-zeta plus polarization orbital basis set, norm-conserving pseudopotentials, an energy cutoff of 200 Rydbergs defined the real space grid and the exchange correlation functional was Local Density Approximation (LDA)^47^.

**Figure 7:**
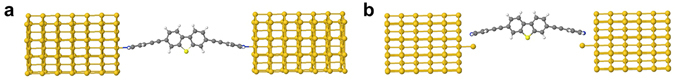
Geometry of the molecular junction containing a 1-S wire (**a**) on flat electrodes (**b**) on electrodes containing adatoms.

**Figure 8:**
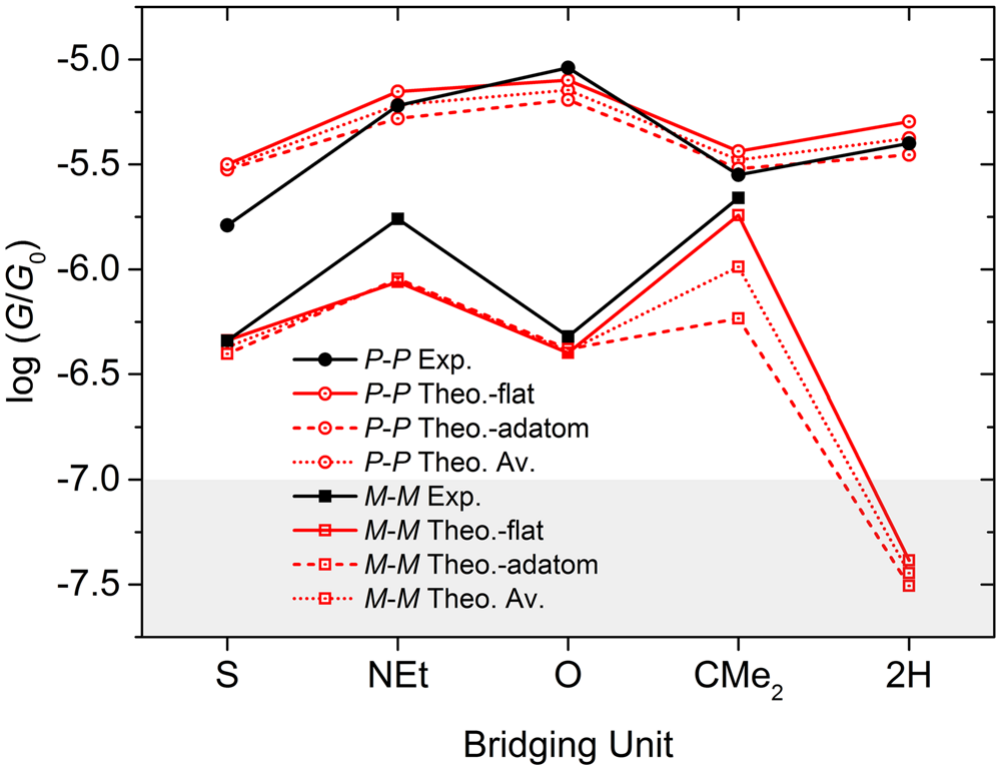
Comparison between theoretical and experimental data for most probable conductance values. DFT results are shown for both flat electrodes (solid red lines) and with electrodes containing adatoms (dashed red lines). The dotted lines show the average of the ‘flat’ and ‘adatom’ DFT conductances. Although there are differences, we conclude that the same qualitative trends are obtained using both geometries, with the exception of CMe_2_, which in the presence of adatoms, no longer has an anomalously high conductance observed experimentally in the *meta* case **9-C** ***m***.

To calculate the conductance through these two groups of molecules, *para* and *meta* shown in Fig. 1, they were attached to gold leads via the pyridyl anchor groups. The leads were constructed of 6 layers of (111) gold each containing 30 gold atoms. Transport calculations were carried out both for flat electrodes and for electrodes containing adatoms, as shown in Fig. 7. According to DFT, the molecule binds most favourably to a top site, with a binding energy of about 0.8 eV at a distance of 2.3 Å (Fig. 7 and Supplementary Figure 34–35) between the terminal nitrogen atoms and a ‘top’ gold atom. This most-favourable binding geometry has been used in all simulations. A Hamiltonian describing this structure was produced using SIESTA and the zero-bias transmission coefficients *T* (E) were calculated using the Gollum code^48^. An excellent agreement between theoretical and experimental conductance values has been obtained (Fig. 8), by choosing a Fermi energy of *E*_F_ = −0.8 eV relative to the DFT-predicted value. Supplementary Figure 49 shows that the transmission coefficients of *meta*-connected molecules are all lower than those of *para* connected molecules over a wide energy range within their HOMO–LUMO gaps, in agreement with a tight-binding model of *pi*-orbital transport (Supplementary Figures 44–48). In the latter case, in the absence of bridging atoms (**5–2H** ***p*** and **10–2H** ***m***) there appear sharp transmission dips due to destructive interference in the *meta* case, which are alleviated by the presence of pendant groups. In contrast, in the *para* case, constructive interference in the *pi*-channel is preserved in the presence of bridging atoms. On the other hand, in the DFT-based transmission curves perfect destructive interference is masked by the presence of sigma orbitals, which provide a parallel path for conductance.

## Discussion

We conclude, therefore, from our experimental and theoretical data for the *para* series **1–4** and the *meta* series **6–9**, that there is a clear correlation between aromaticity of the central ring when heteroatoms are present and the single-molecule conductance value in the *para* series. Aromaticity follows the sequence: S > NEt > O > CMe_2_. Our conductance trend for the heterocyclic *para* series, i.e. O > NEt > S is in agreement with a previous experimental study by Venkataraman, Breslow *et al*.^24^ on monocyclic core units (furan > thiophene) (Fig. 2). However, the fluorene derivative **4-C** ***p*** (which does not have a heteroatom in the core) shows a lower conductance than **2-N** ***p*** and **3-O** ***p*** although it exhibits a non-aromatic core. The reason for this exceptional behaviour of **4-C** ***p*** is not clear. However, we note that other workers^21–23^ have observed that the single-molecule conductance of fluorene-based cores do not follow expected trends.

These results demonstrates that a non-aromatic core unit does not necessarily lead to higher conductance since the polycyclic series **1–5** exhibits a clear difference in comparison to the monocyclic series shown in Scheme 2. However, our data show that the sequence is very different in the *meta* series where dibenzothiophene ≈ dibenzofuran < carbazole. Multiple factors (such as quantum interference, aromaticity and electronegativity) and their composite effects should be taken into consideration in explaining the trends in the conductance. Our results show that bridging heteroatoms alleviate destructive quantum interference in the *meta*-connected molecules. The contribution of electronegativity of the bridging atoms should not be ignored. For the *meta* series dibenzothiophene **6-S** ***m*** and dibenzofuran **8-O** ***m*** represent both extremes. Dibenzothiophene **S** ***m*** is the most aromatic and therefore it lowers the conductance. As was mentioned above, dibenzofuran **8-O** ***m*** is the least aromatic core unit. Therefore, based on the conclusions of the series shown in Fig. 2, **8-O** ***m*** should be the most conductive molecule. However, the lone pair of oxygen is tightly bonded due to oxygen’s high electronegativity, which hinders the delocalization of electrons and decreases the electron density of the conjugated π system in **8-O** ***m***. The carbazole derivative **7-N** ***m*** is the most conductive in this *meta* series because it is less aromatic than dibenzothiophene, but also bears a lone pair which allows transmission through the molecule. The conductance of model non-bridged compound **5–2H** ***p*** is reduced because of the dihedral angle between the two phenyl units. Model compound **10–2H** ***m*** shows no conductance within the detection limit of the MCBJ setup. This is consistent with the bridging atom of **6–9** planarizing the core, which is essential for raising the conductance in the *meta*-series.

We have studied the single-molecule conductance of ten oligo(arylene-ethynylene) derivatives with five different core units (dibenzothiophene, carbazole, dibenzofuran, fluorene and biphenyl) attached to gold electrodes by pyridyl anchoring groups. Within the two series there is either *para-para* or *meta-meta* conjugation through the core unit. In all cases molecules with *para* connectivity present larger conductances than their *meta* isomers, regardless of the bridging unit. We have experimentally and theoretically observed clear and distinct trends in the *para* and *meta* series. In the *para* series there is a clear correlation between aromaticity of the central ring and the single-molecule conductance values in the sequence dibenzofuran > carbazole > dibenzothiophene, in agreement with a previous experimental study on monocyclic core units (furan > thiophene). However, in the *meta* series the carbazole derivative is the most conductive: the sequence dibenzothiophene ≈ dibenzofuran < carbazole. It is concluded that the nitrogen lone pair facilitates transmission through the molecule. Overall, we find that constructive quantum interference in the *para*-connected molecules persists in the presence of bridging atoms and is partly masked by the presence of sigma channels, whereas bridging atoms alleviate destructive quantum interference in the *meta*-connected molecules. Our comprehensive study establishes that both quantum interference and heteroaromaticity in the molecular core units play important and inter-related roles in determining the conductance of single molecular junctions. These results should assist in future research in the development of new molecules for incorporation into nanoscale molecular circuits.

## Methods

### Synthesis

Details of the synthesis and molecular characterization are in the Supplementary Notes 1–2. Synthetic procedures, characterization data; UV–Vis absorption; NMR spectra; and MCBJ conductance analysis of compounds 1–10; additional theoretical data.

### MCBJ measurements

Electron transport characteristics in single-molecule junctions were studied by MCBJ measurements in solution at room temperature. The molecular solution contained typically 0.1 mM of the **1–10** molecules in a mixture of 1,3,5-trimethylbenzene (Aldrich, p.a.) and tetrahydrofuran (Aldrich, p.a.), 4:1 (v/v). Single-molecule conductance experiments were performed during the formation and breaking of a nanogap on a notched, freely suspended gold wire (0.1 mm diameter, 99.999%, Goodfellow) fixed on spring steel sheets (10 mm × 30 mm, 0.25 mm thick) with a two-component epoxy glue (Stycast 2850 FT with catalyst 9). A Kel-F liquid cell with a Kalrez O-ring was mounted onto the sample sheet fixed by two holders. During the measurements, the steel sample could be bent with a pushing rod controlled by a stepper motor (or a piezo motor) and a piezo stack. The stepper motor initialized the bending process. Once the measured current reached a value corresponding to 15 *G*_0_, the stepper motor paused and the piezo stack was activated. This strategy managed to decrease environmental noise significantly from the operation of the stepper motor. After the junction was completely opened, the piezo stack was reset and the stepper motor drove down the pushing rod. The movement of the piezo stack controlled the breaking and the reformation of nanoscale contacts and the stretching rate of the two gold leads controlled by the piezo stack is about 5–20 nm/s. Molecular junctions could form upon breaking the gold–gold nanocontacts. More than 1000 conductance–distance curves were recorded for statistically relevant data analysis during the repeated cycles.

The MCBJ controller is based on a laboratory-built bipotentiostat. All current measurements were performed with two custom-designed bipolar and tunable logarithmic I–V converters operating in a wide dynamic range from 10 to below 10^−7^ *G*_0_. The tunneling current between the two ends of the ‘broken wire’ (taken as WE1 and WE2) could be recorded as a feedback signal at a given bias voltage 0.1 V. The distance between the two gold electrodes in the MCBJ setup is calibrated by the STM-BJ setup with the assumption that the tunneling decay is identical under the same experimental conditions. After breaking a gold-gold contact, the conductance of the junction drops to approximately 10^−3^ *G*_0_. Due to the so-called “snap-back” effect, the gap between the two gold electrodes increases instantaneously to a certain distance Δ*z*_corr_. A perfect linear atomic chain of gold has a conductance of *G*_0_. Due to the tunneling theory, we assume that log (G/*G*_0_) = −α*z*_exp_, where *z*_exp_ = 0 corresponds to the point where the distance between the terminating gold atoms is equal to the equilibrium gold-gold separation. Our measured separation is Δ*z* = *z*_exp_ − Δ*z*_corr_, where Δ*z*_corr_ is the snap-back distance. Then, the relationship between log (G/*G*_0_) and Δ*z* is log (G/*G*_0_) = −αΔ*z* − αΔ*z*_corr_. The log(G/*G*_0_) plot has a slope of −α and an intercept of −αΔ*z*_corr_. To calibrate the stretching distance in the absence of molecules, we measured the conductance G versus Δ*z* for conductances ranging from 10^−4^ to 10^−6^ *G*_0_ and extracted the slope and intercept. From repeated measurements, we obtained a distribution of slopes and intercepts and from the most probable slopes and intercepts obtained the most probable values of α and Δ*z*_corr_. We concluded from individual experiments that in TMB: THF (v:v = 4:1), the Δ*z*_corr_ is determined as 0.5 nm and the addition of a low concentration of molecules in solution does not influence the snap-back distance. Further technical details and data evaluation methods have been described in our previous article by Hong *et al*.^49^.

### Theory and simulations

Details of the theory and simulations are in Supplementary Notes 4–5.

## Electronic supplementary material

SI
