# Supplementary material for: Quantum interference and heteroaromaticity of para- and meta-linked bridged biphenyl units in single molecular conductance measurements
Source: Sci Rep. 2017 May 11;7:1794. doi: 10.1038/s41598-017-01903-0 (PMC5431762; doi:10.1038/s41598-017-01903-0)
Supplement: Supplementary file 1 — SI [file 41598_2017_1903_MOESM1_ESM.pdf]

# Supplementary Information

## Quantum interference and heteroaromaticity of *para*- and *meta*-linked bridged biphenyl units in single molecular conductance measurements

Markus Gantenbein[1], Lin Wang[2,7,8], Alaa A. Al-jobory[3,4], Ali K. Ismael[3,5], Colin J. Lambert[3], Wenjing Hong[2,6] & Martin R. Bryce[1]

[1]Department of Chemistry, Durham University, Durham DH1 3LE, UK.

[2]Department of Chemistry and Biochemistry, University of Bern, Freiestrasse 3, Bern CH-3012, Switzerland.

[3]Department of Physics, Lancaster University, Lancaster LA1 4YB, UK.

[4]Department of Physics, College of Education for Pure Science, Anbar University, Anbar, Iraq.

[5]Department of Physics, College of Education for Pure Science, Tikrit University, Tikrit, Iraq.

[6]Collaborative Innovation Center of Chemistry for Energy Materials, Department Chemical and Biochemical Engineering, College of Chemistry and Chemical Engineering, Xiamen University, Xiamen, China 3610054.

[7]Key Laboratory of Molecular Nanostructure and Nanotechnology, Institute of Chemistry Chinese Academy of Sciences, Beijing, China 100190.

[8]University of the Chinese Academy of Sciences, Beijing, China.

Correspondence: M.R.B. (email: m.r.bryce@durham.ac.uk), C.J.L. (email: c.lambert@lancaster.ac.uk), W.H. (email: whong@xmu.edu.cn)

## Supplementary Figures

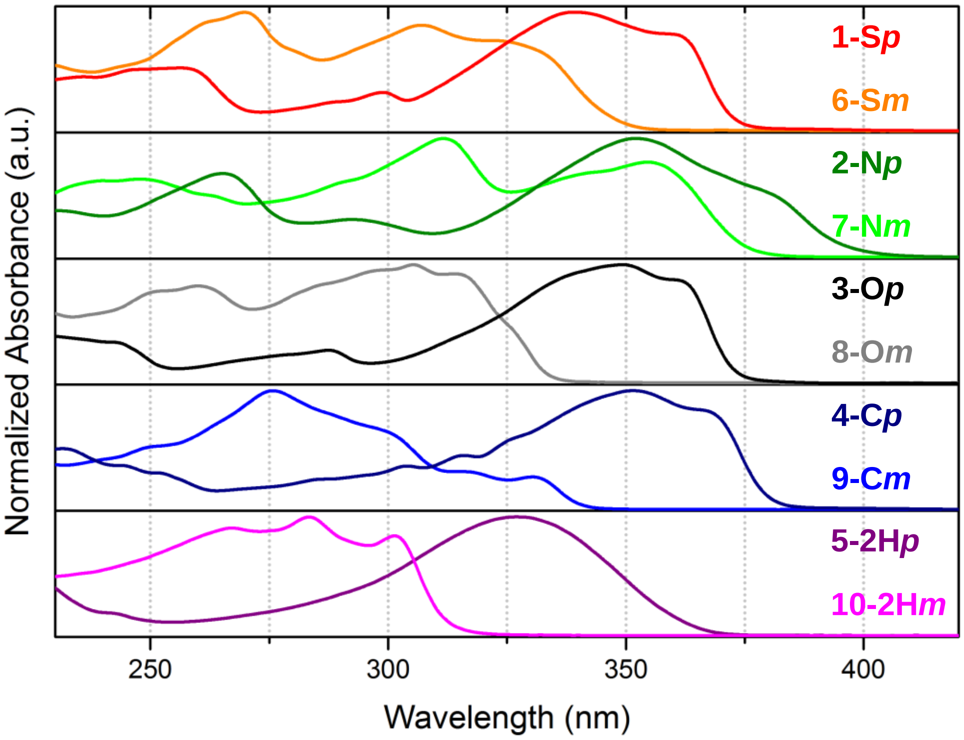


**Supplementary Figure 1.** UV–Vis absorption spectra of compounds **1–10** in a dichloromethane solution.

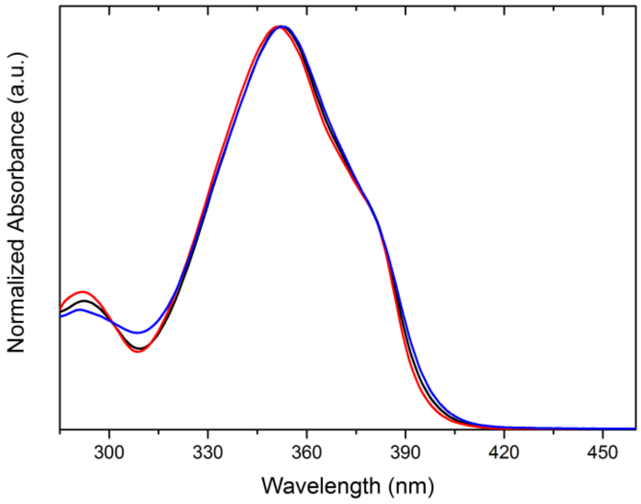


**Supplementary Figure 2.** UV–Vis absorption spectra of compound **2-N*p*** in dichloromethane (black), toluene (red), and ethanol (blue).

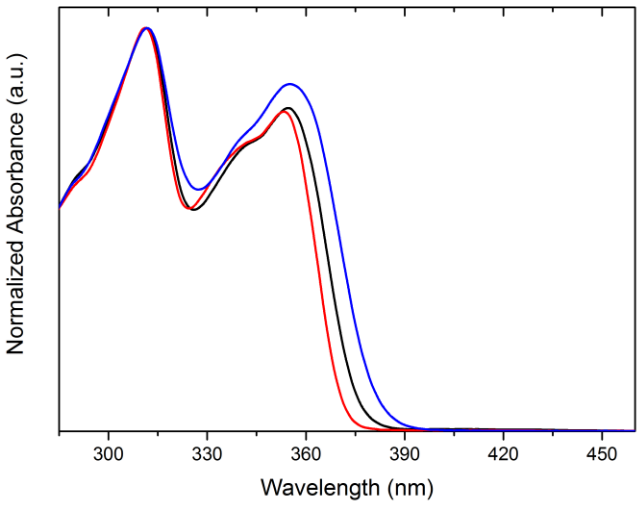


**Supplementary Figure 3.** UV–Vis absorption spectra of compound **7-N*m*** in dichloromethane (black), toluene (red), and ethanol (blue).

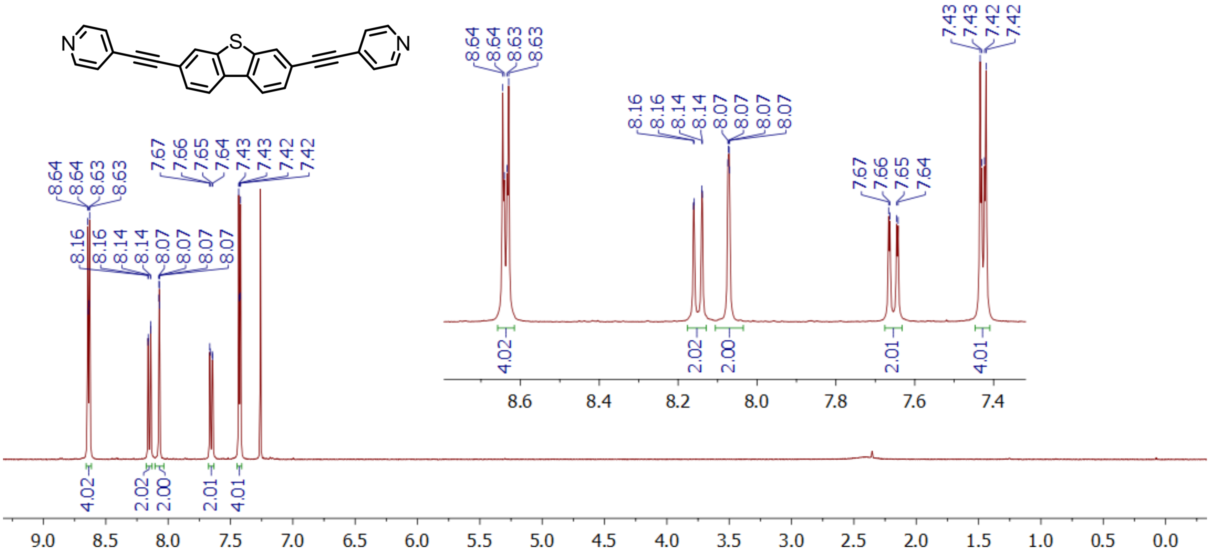


**Supplementary Figure 4.** $^{1}$H NMR Spectrum of **1-S*p*** in $CDCl_3$ ($\delta$ $CHCl_3$ 7.26)

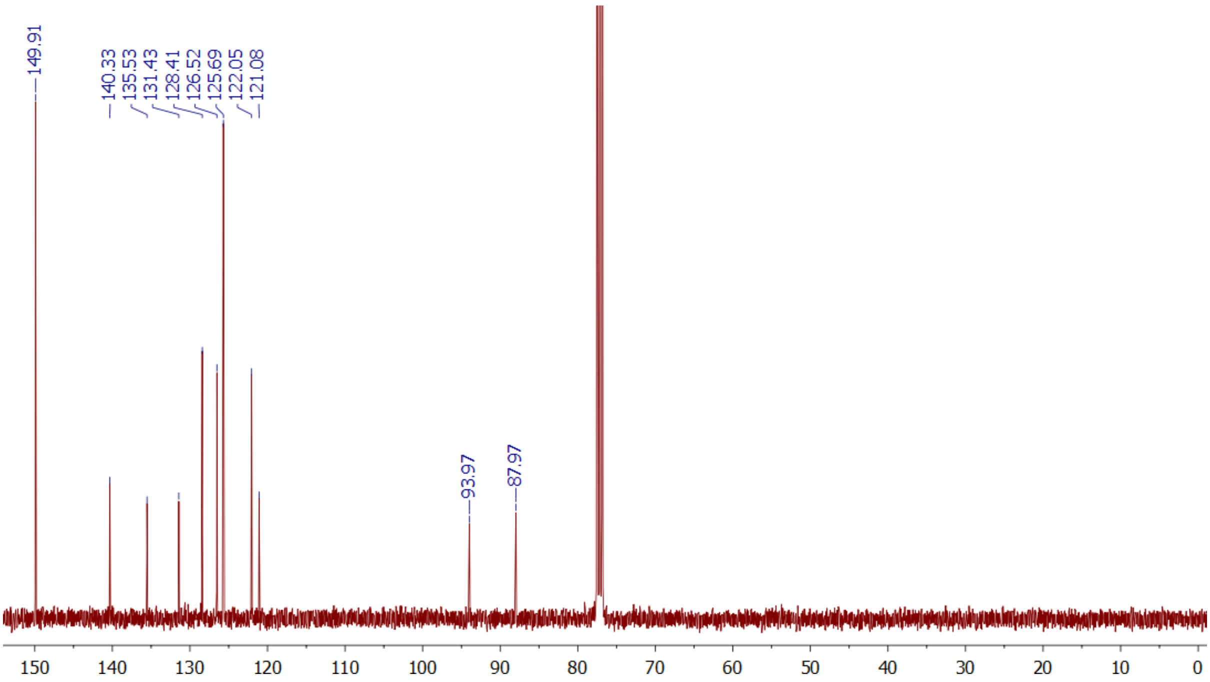


**Supplementary Figure 5.** $^{13}$C NMR Spectrum of **1-S*p*** in $CDCl_3$ ($\delta$ 77.48, 77.16, 76.84)

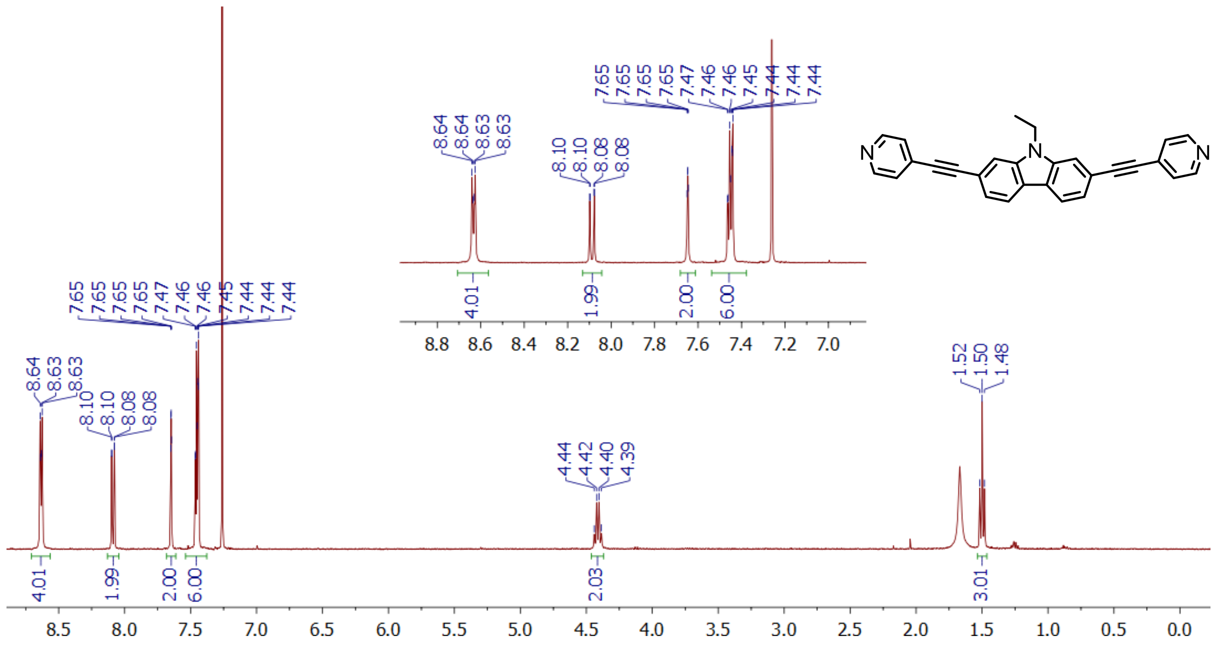

**Supplementary Figure 6.** $^{1}$H NMR Spectra of **2-N*p*** in $CDCl_3$ ($\delta$ $CHCl_3$ 7.26)

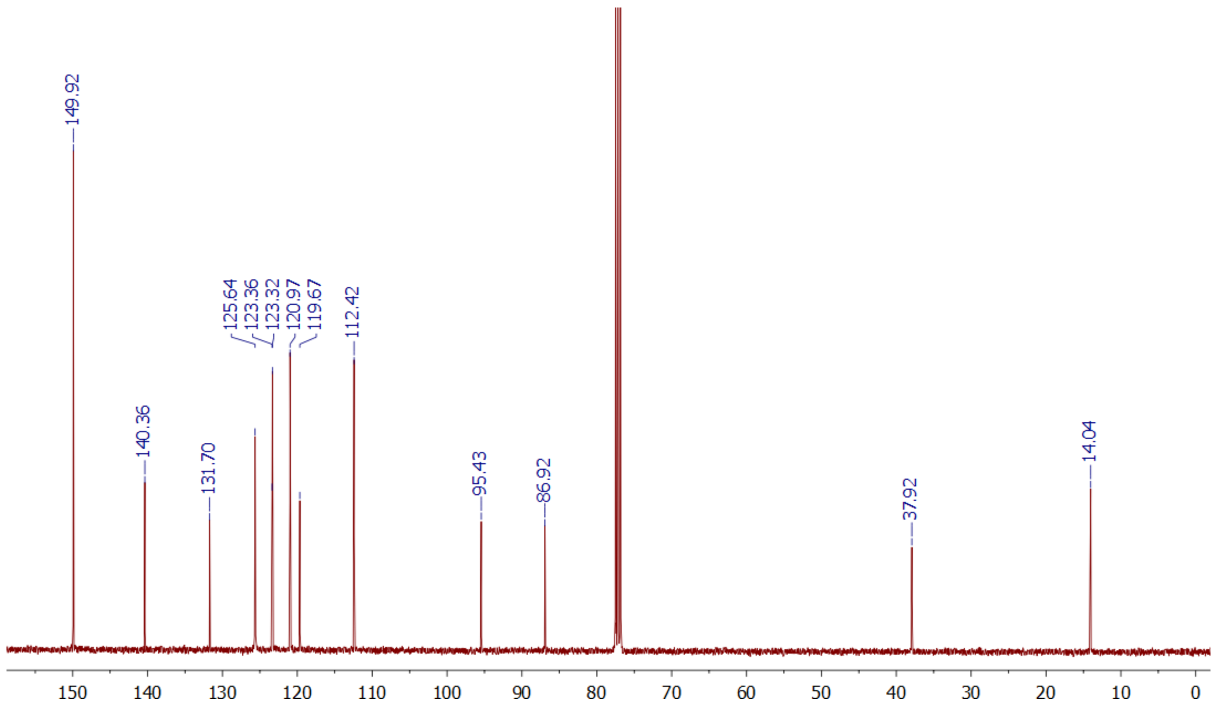

**Supplementary Figure 7.** $^{13}$C NMR Spectrum of **2-N*p*** in $CDCl_3$ ($\delta$ 77.48, 77.16, 76.84)

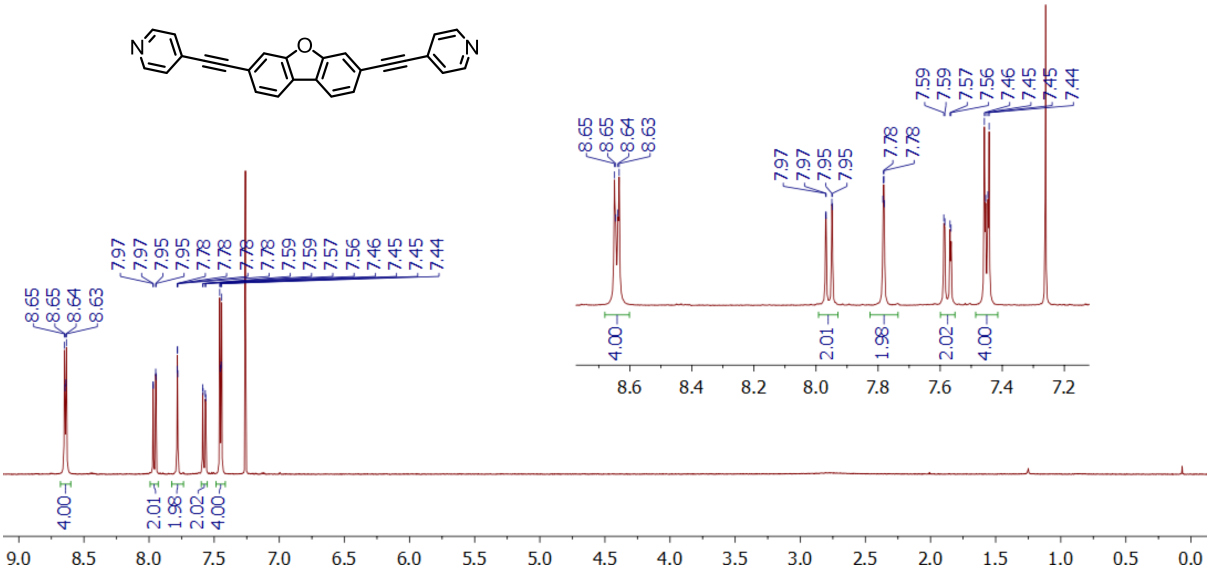


**Supplementary Figure 8.** $^{1}$H NMR Spectrum of **3-O*p*** in $CDCl_3$ ($\delta$ $CHCl_3$ 7.26)

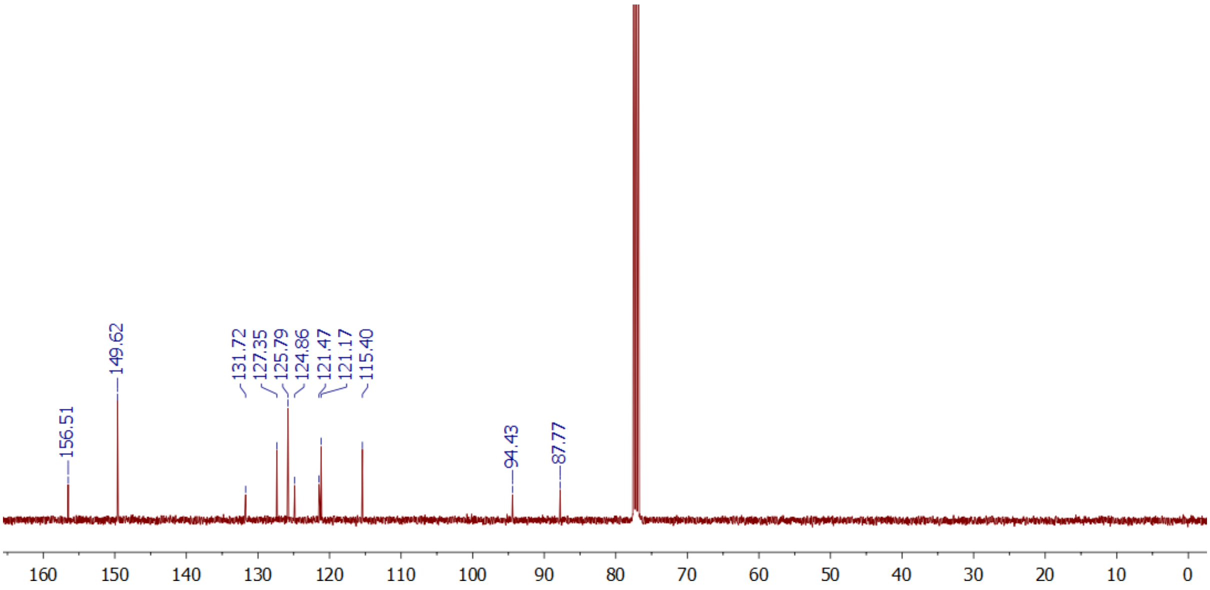


**Supplementary Figure 9.** $^{13}$C NMR Spectrum of **3-O*p*** in $CDCl_3$ ($\delta$ 77.48, 77.16, 76.84)

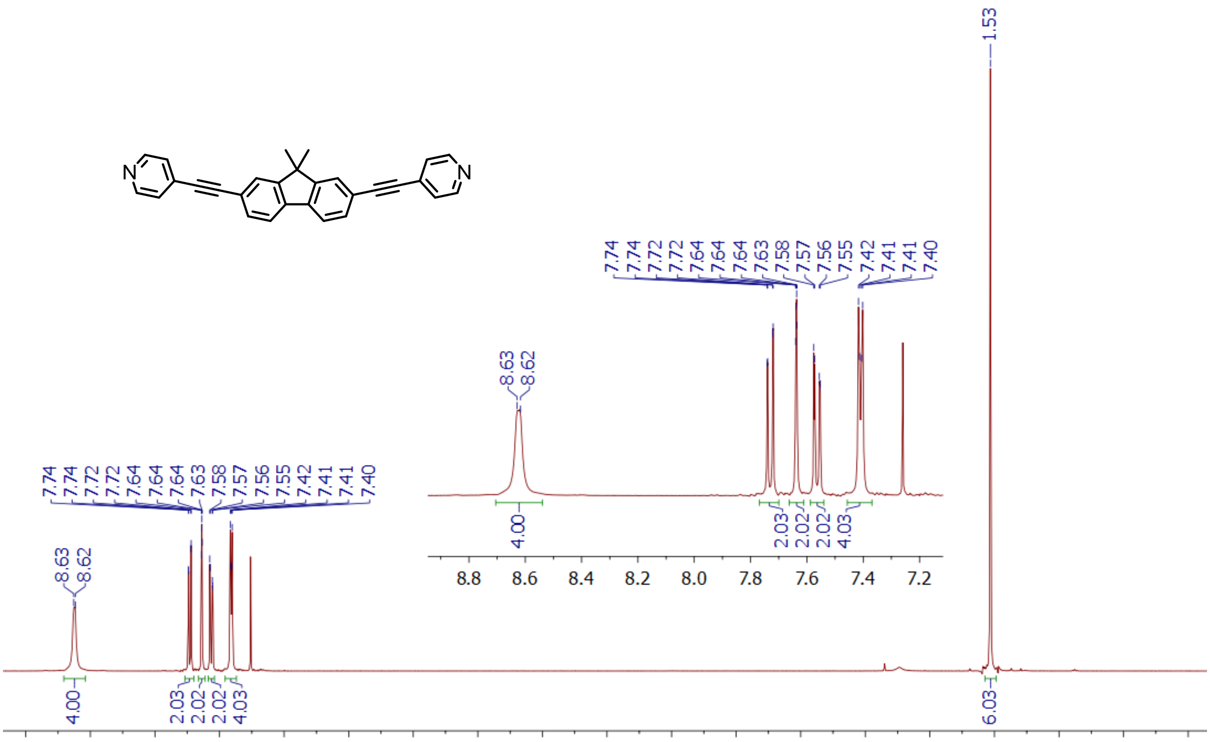


**Supplementary Figure 10.** $^{1}$H NMR Spectrum of **4-C*p*** in $CDCl_3$ ($\delta$ $CHCl_3$ 7.26)

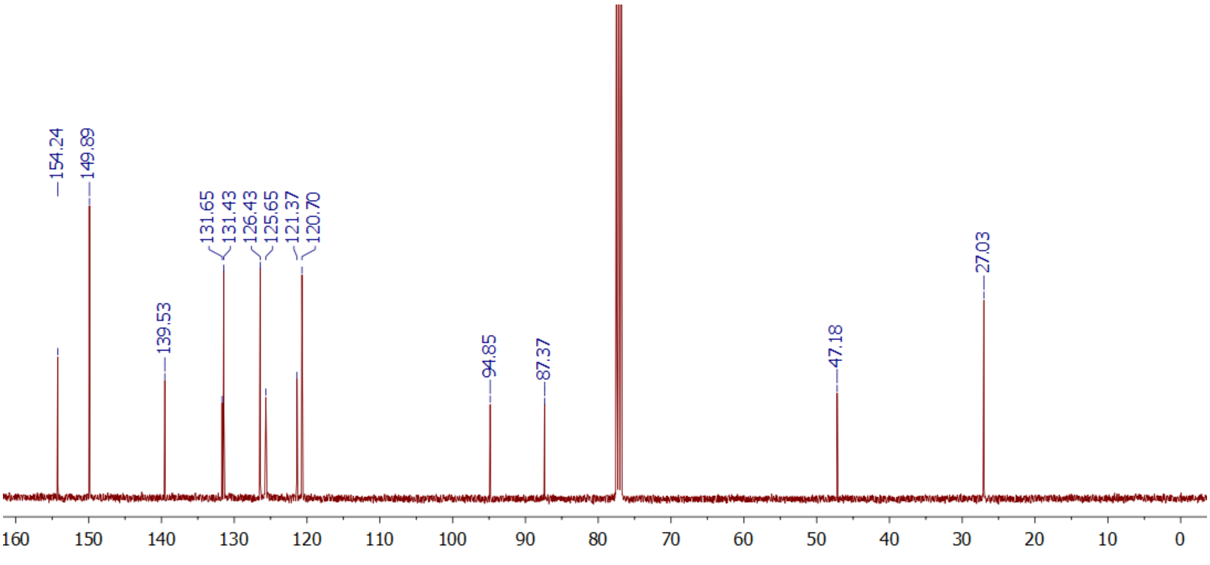


**Supplementary Figure 11.** $^{13}$C NMR Spectrum of **4-C*p*** in $CDCl_3$ ($\delta$ 77.48, 77.16, 76.84)

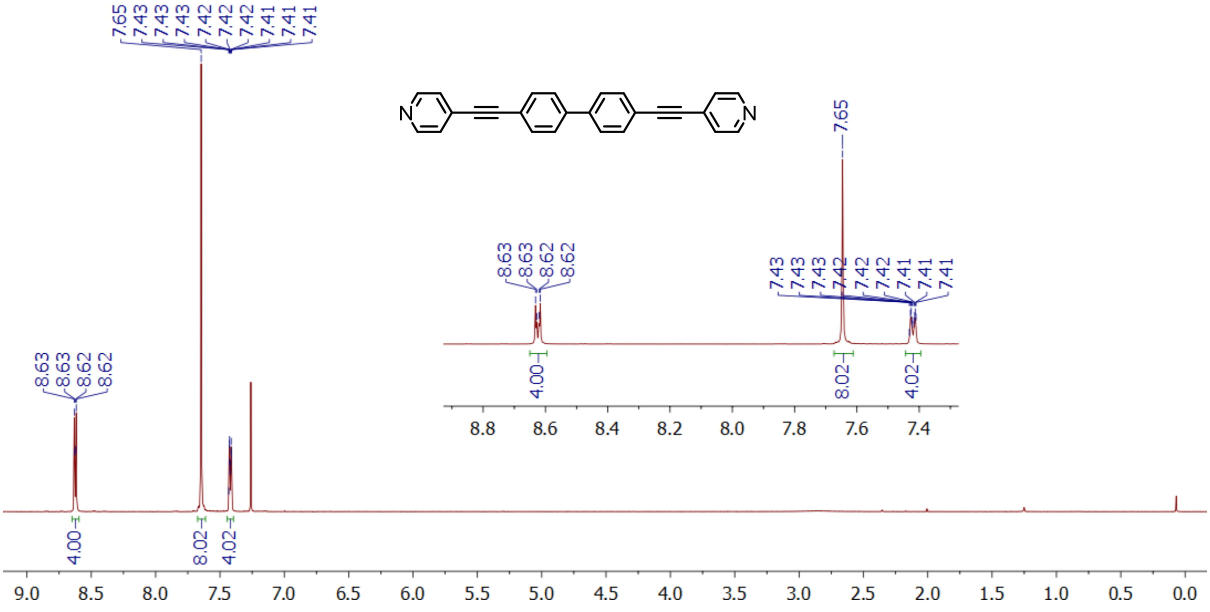


**Supplementary Figure 12.** $^{1}$H NMR Spectrum of **5-2H*p*** in $CDCl_3$ ($\delta$ $CHCl_3$ 7.26)

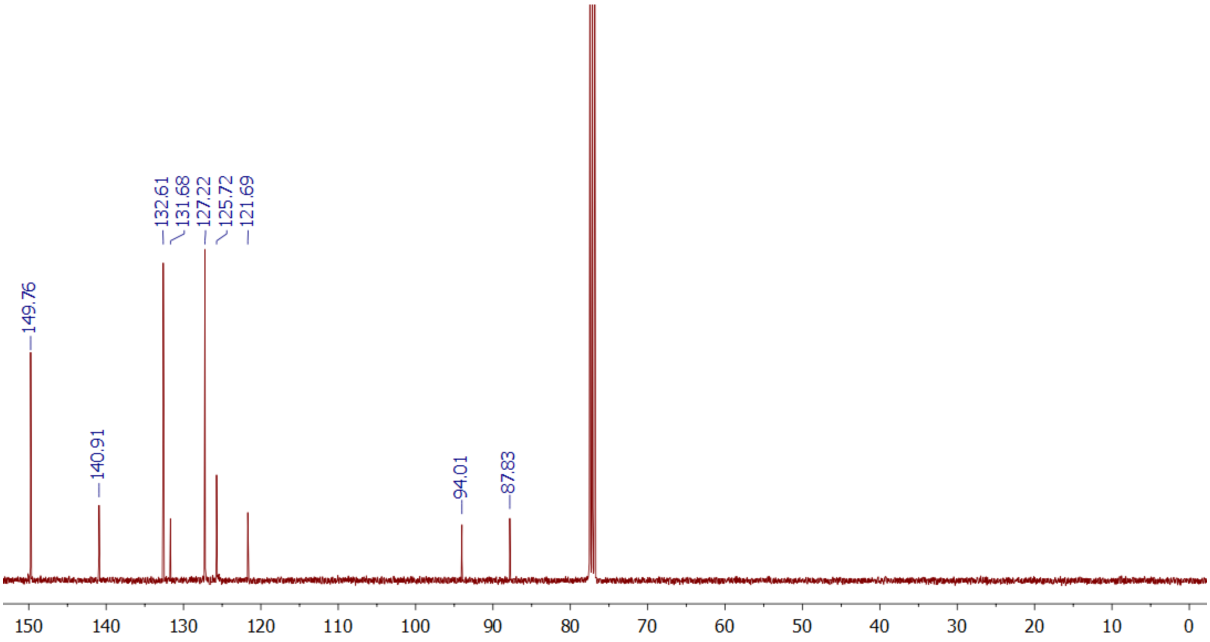


**Supplementary Figure 13.** $^{13}$C NMR Spectrum of **5-2H*p*** in $CDCl_3$ ($\delta$ $CHCl_3$ 77.48, 77.16, 76.84)

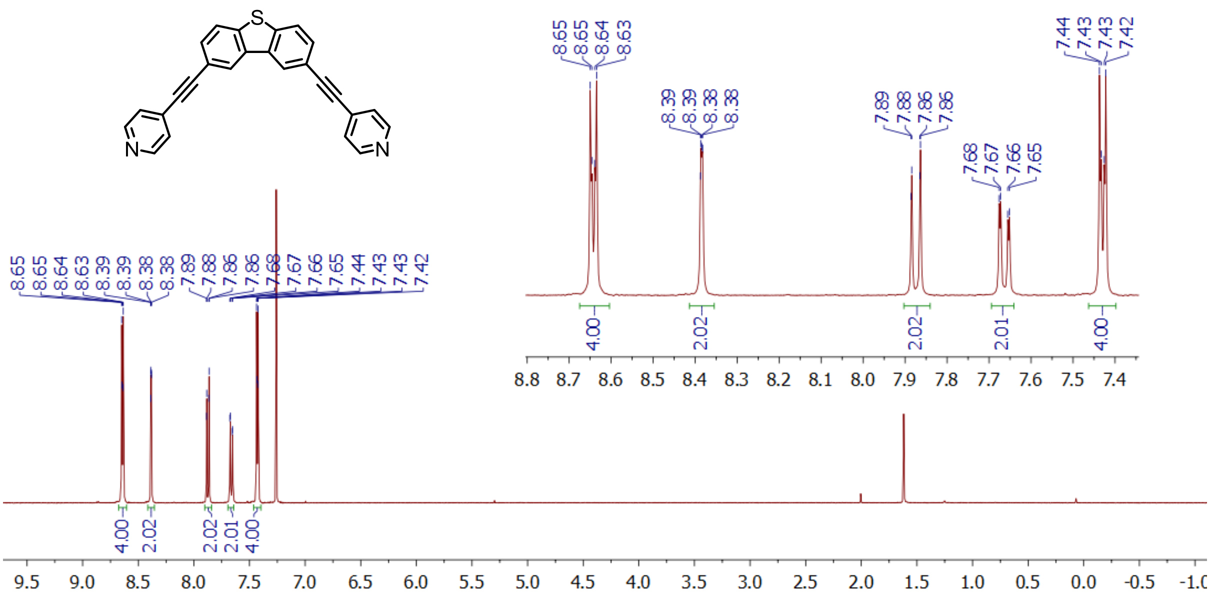


**Supplementary Figure 14.** $^{1}$H NMR Spectrum of **6-S*m*** in $CDCl_3$ ($\delta$ $CHCl_3$ 7.26)

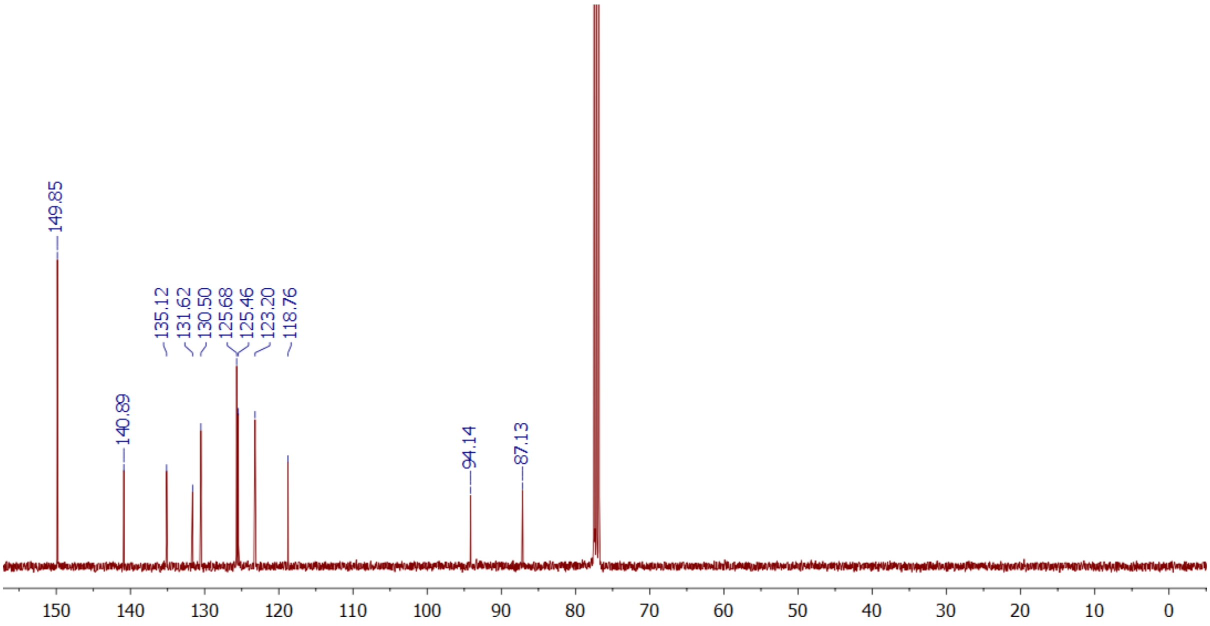


**Supplementary Figure 15.** $^{13}$C NMR Spectrum of **6-S*m*** in $CDCl_3$ ($\delta$ 77.48, 77.16, 76.84)

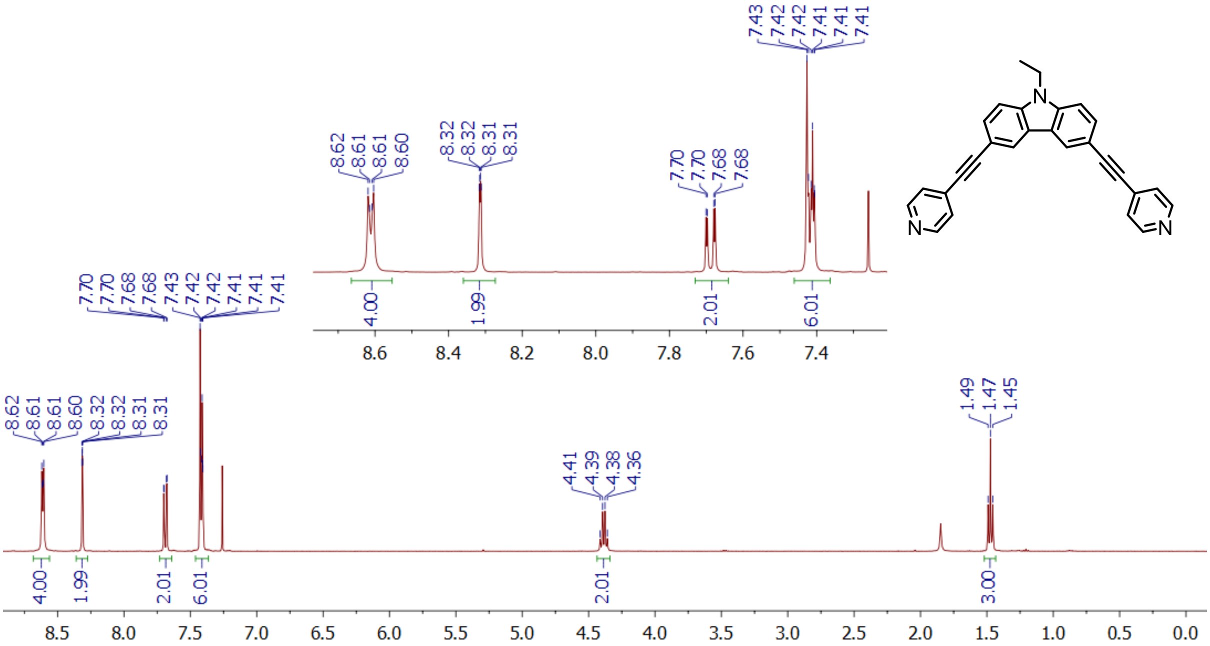


**Supplementary Figure 16.** $^{1}$H NMR Spectrum of **7-N*m*** in $CDCl_3$ ($\delta$ $CHCl_3$ 7.26)

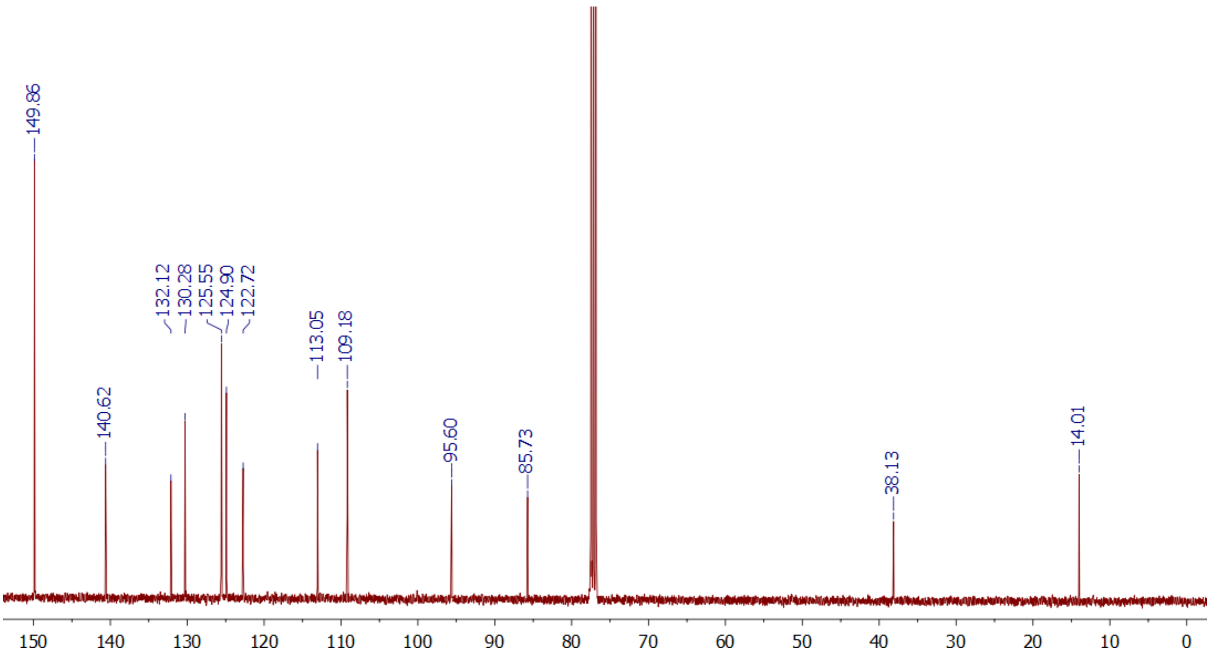


**Supplementary Figure 17.** $^{13}$C NMR Spectrum of **7-N*m*** in $CDCl_3$ ($\delta$ 77.48, 77.16, 76.84)

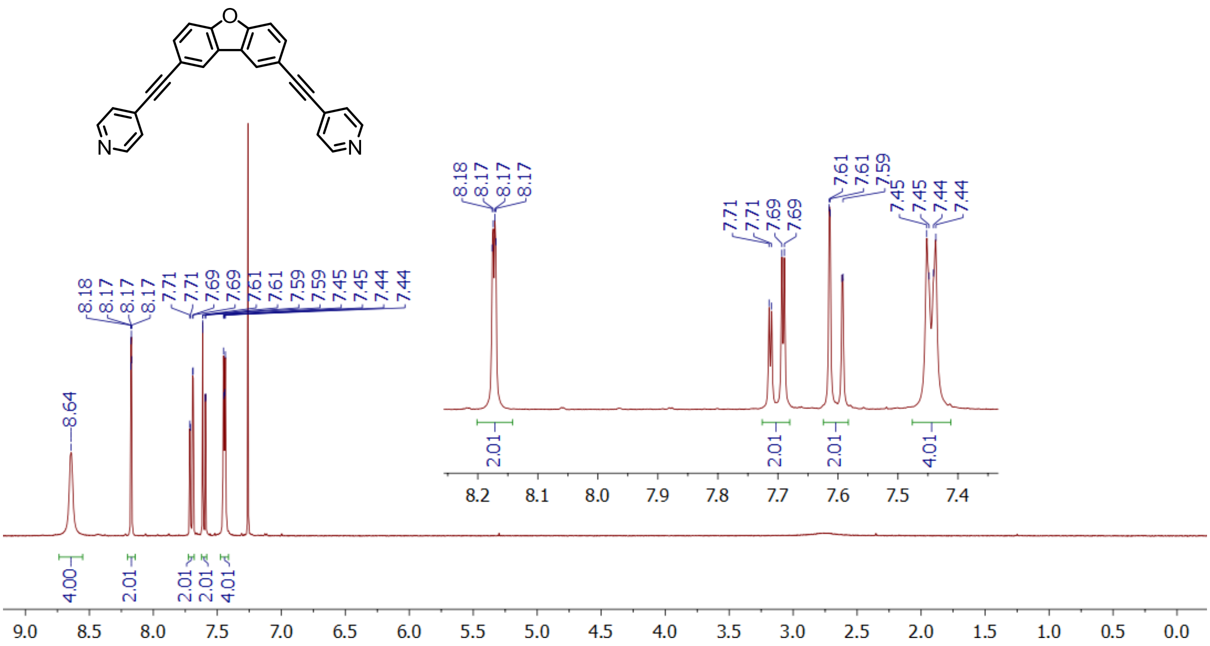


**Supplementary Figure 18.** $^{1}$H NMR Spectrum of **8-O*m*** in $CDCl_3$ ($\delta$ $CHCl_3$ 7.26)

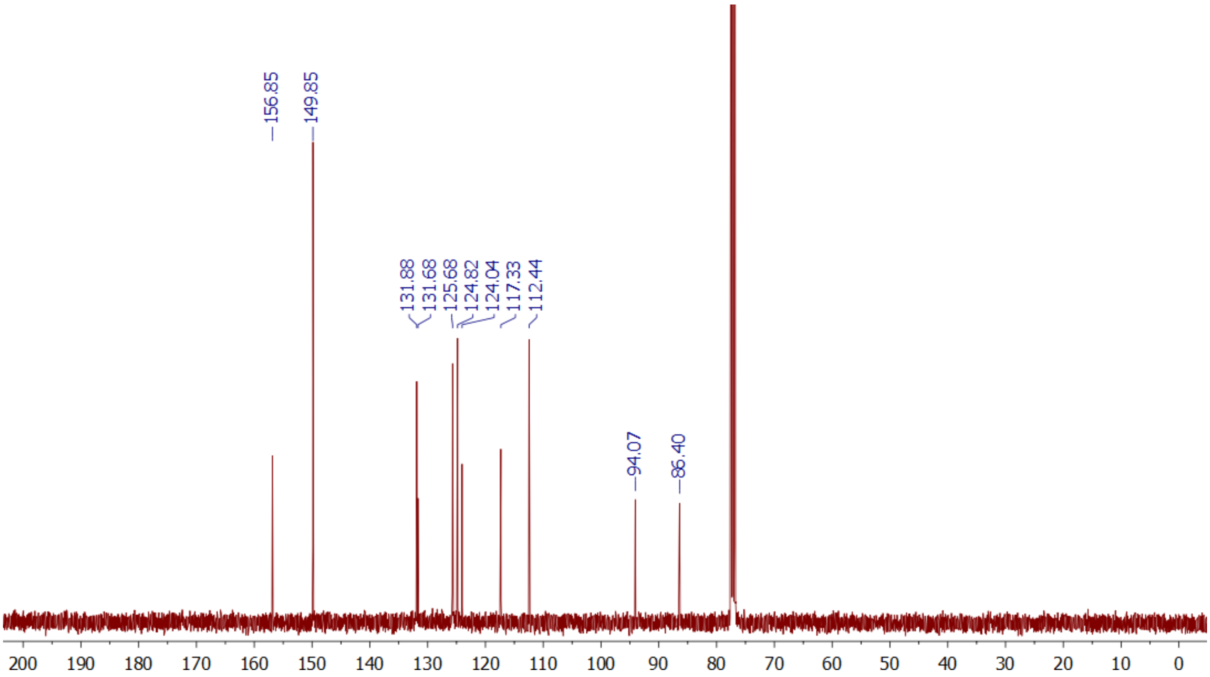


**Supplementary Figure 19.** $^{13}$C NMR Spectrum of **8-O*m*** in $CDCl_3$ ($\delta$ 77.48, 77.16, 76.84)

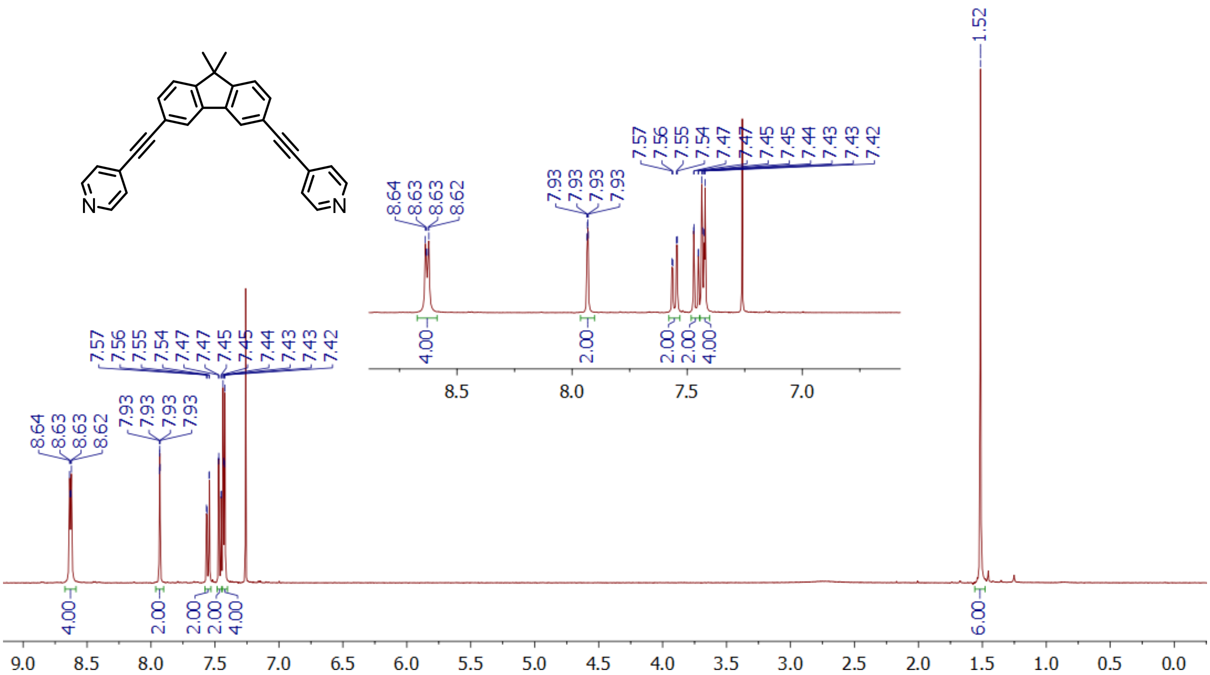


**Supplementary Figure 20.** $^{1}$H NMR Spectrum of **9-C*m*** in $CDCl_3$ ($\delta$ $CHCl_3$ 7.26)

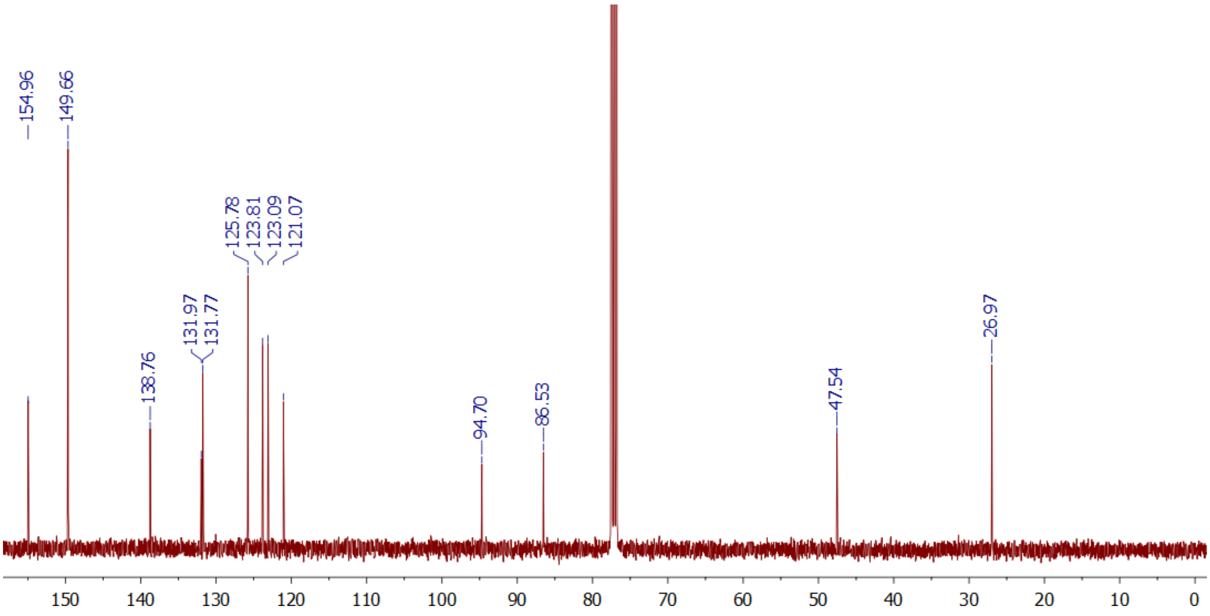


**Supplementary Figure 21.** $^{13}$C NMR Spectrum of **9-C*m*** in $CDCl_3$ ($\delta$ 77.48, 77.16, 76.84)

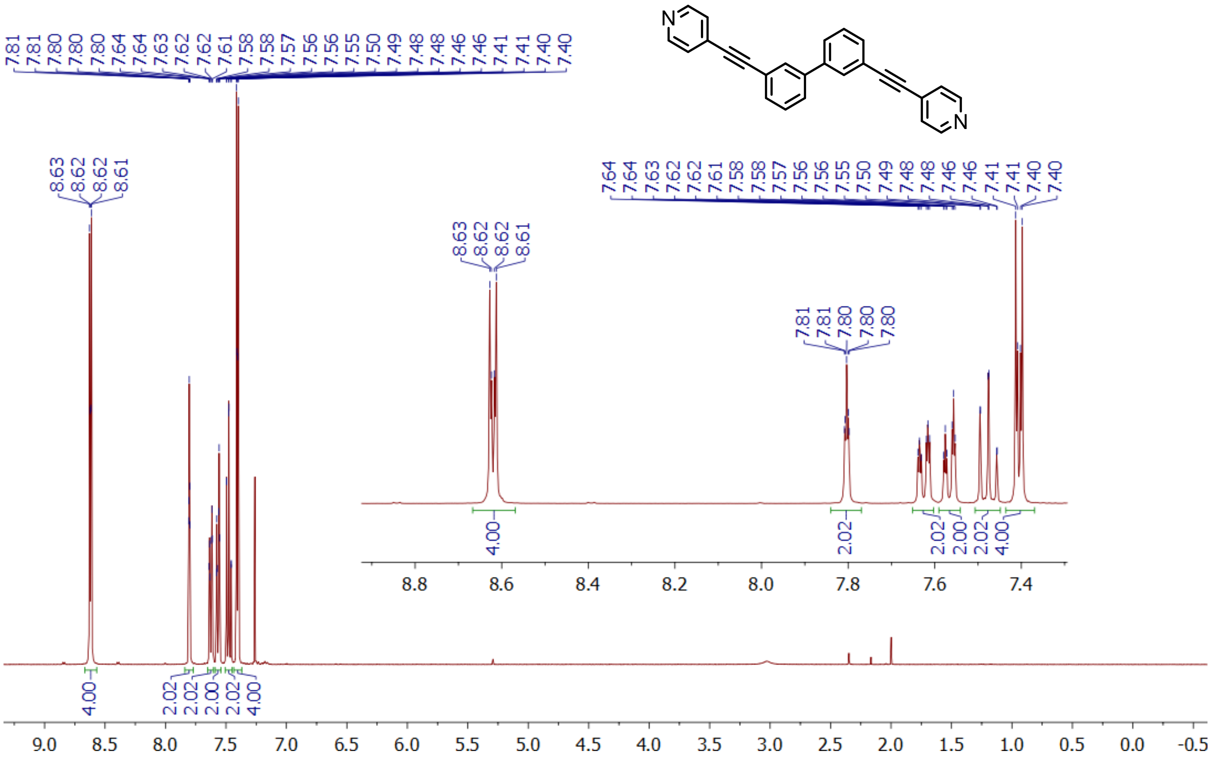


**Supplementary Figure 22.** $^{1}$H NMR Spectrum of **10-2H*m*** in $CDCl_3$ ($\delta$ $CHCl_3$ 7.26)

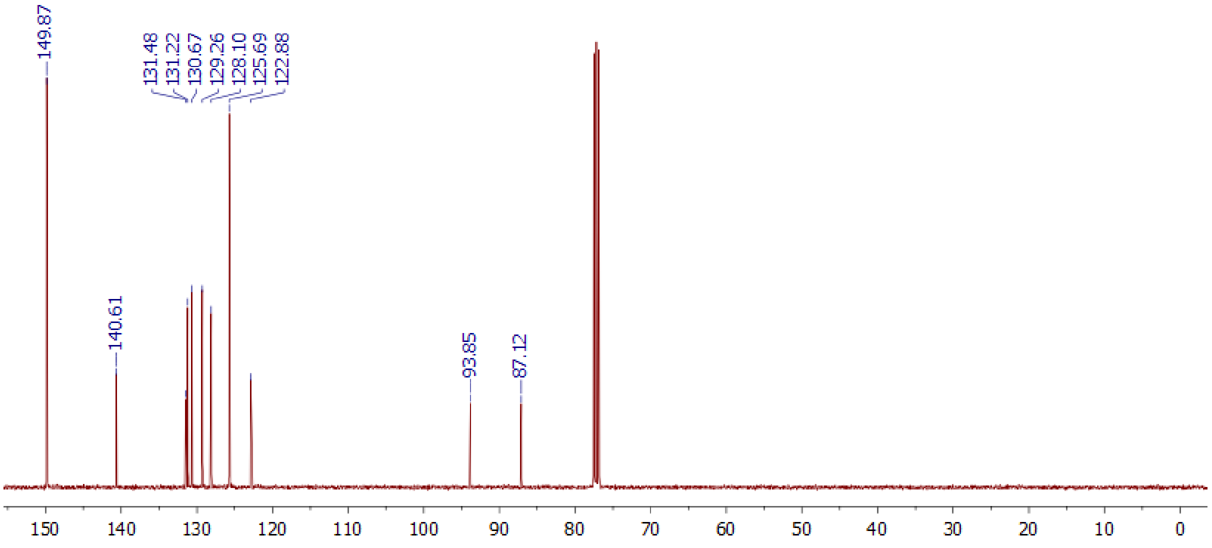


**Supplementary Figure 23.** $^{13}$C NMR Spectrum of **10-2H*m*** in $CDCl_3$ ($\delta$ 77.48, 77.16, 76.84)

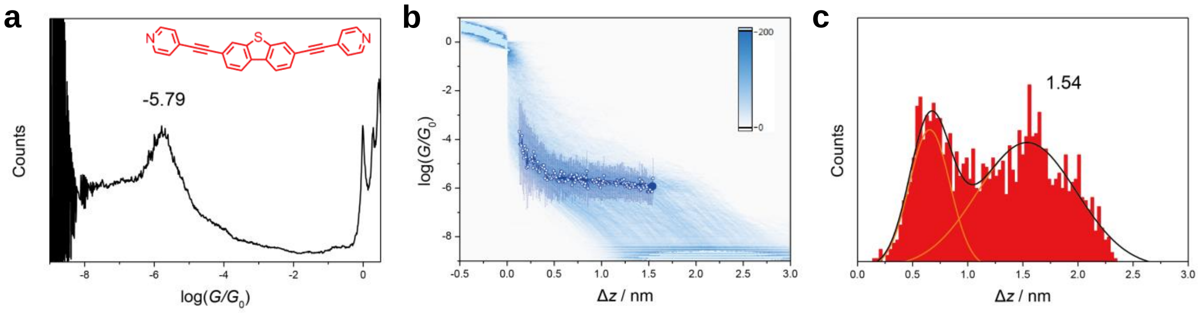


**Supplementary Figure 24.** (**a**) 1D conductance histograms, (**b**) 2D histogram and (**c**) characteristic plateau length distribution between $10^{-0.30}$ $G_0$ and $10^{-6.51}$ $G_0$ in MCBJ experiments of molecule **1-S*p***. In the 2D histogram, statistically averaged conductance−distance traces (hollow circles) with variations indicated by the standard deviations (bars) are shown, along with the linear fit (line). The solid circle represents the last data point in the linear fit before junction rupture.

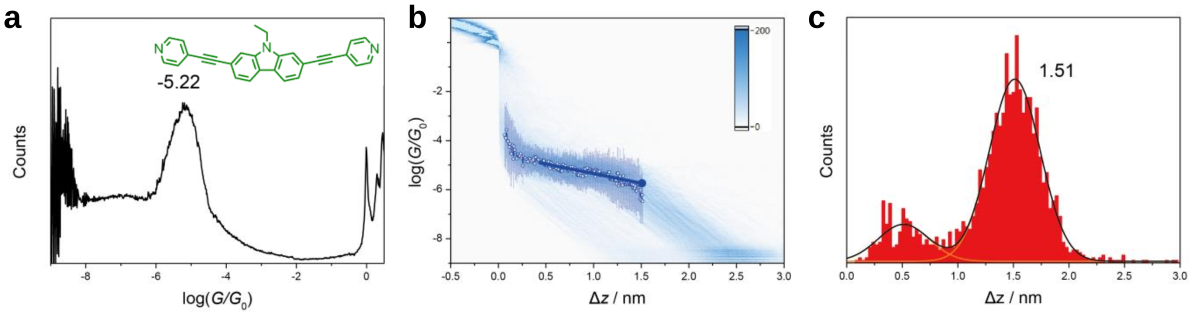


**Supplementary Figure 25.** (**a**) 1D conductance histogram, (**b**) 2D histogram and (**c**) characteristic plateau length distribution between $10^{-0.30}$ $G_0$ and $10^{-6.25}$ $G_0$ in MCBJ experiment of molecule **2-N*p***. In the 2D histogram, statistically averaged conductance−distance traces (hollow circles) with variations indicated by the standard deviations (bars) are shown, along with the linear fit (line). The solid circle represents the last data point in the linear fit before junction rupture.

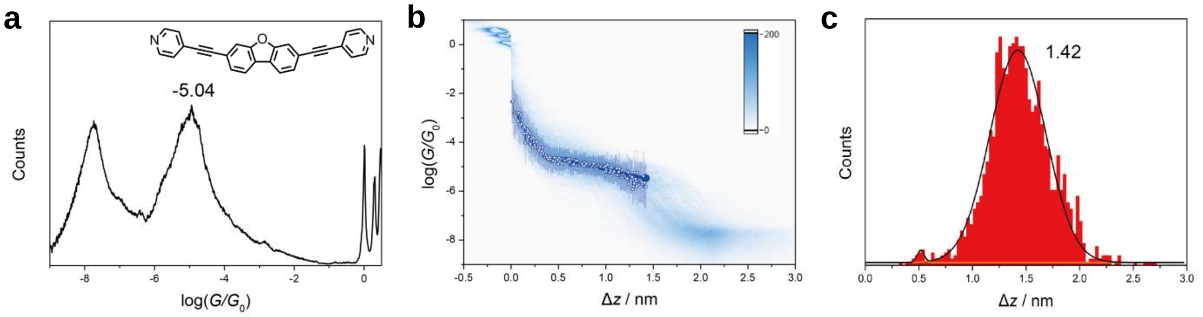


**Supplementary Figure 26.** (**a**) 1D conductance histogram, (**b**) 2D histogram and (**c**) characteristic plateau length distribution between $10^{-0.30}$ $G_0$ and $10^{-6.29}$ $G_0$ in MCBJ experiment of molecule **3-O*p***. In the 2D histogram, statistically averaged conductance–distance traces (hollow circles) with variations indicated by the standard deviations (bars) are shown, along with the linear fit (line). The solid circle represents the last data point in the linear fit before junction rupture.

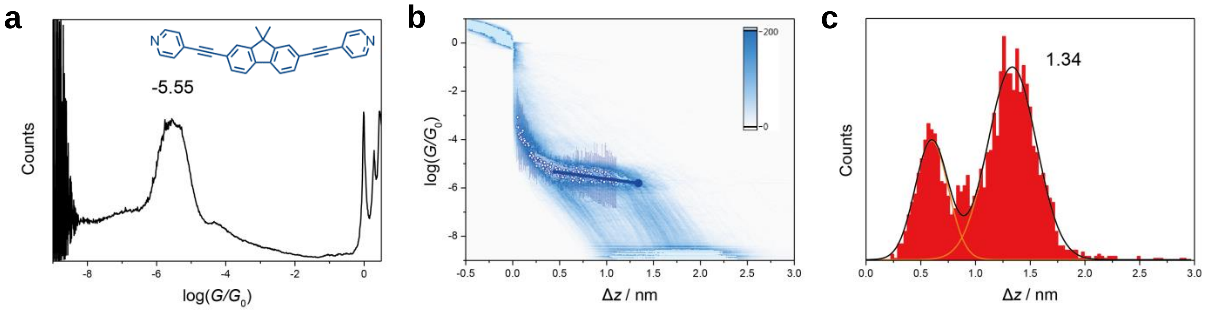


**Supplementary Figure 27.** (**a**) 1D conductance histogram, (**b**) 2D histogram and (**c**) characteristic plateau length distribution between $10^{-0.30}$ $G_0$ and $10^{-6.47}$ $G_0$ in MCBJ experiment of molecule **4-C*p***. In the 2D histogram, statistically averaged conductance–distance traces (hollow circles) with variations indicated by the standard deviations (bars) are shown, along with the linear fit (line). The solid circle represents the last data point in the linear fit before junction rupture.

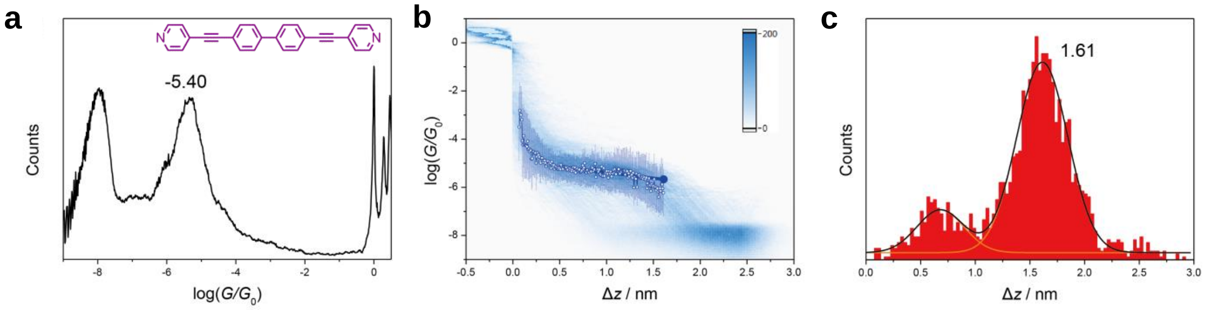


**Supplementary Figure 28.** (**a**) 1D conductance histogram, (**b**) 2D histogram and (**c**) characteristic plateau length distribution between $10^{-0.30}$ $G_0$ and $10^{-6.51}$ $G_0$ in MCBJ experiment of molecule **5-2H*p***. In the 2D histogram, statistically averaged conductance−distance traces (hollow circles) with variations indicated by the standard deviations (bars) are shown, along with the linear fit (line). The solid circle represents the last data point in the linear fit before junction rupture.

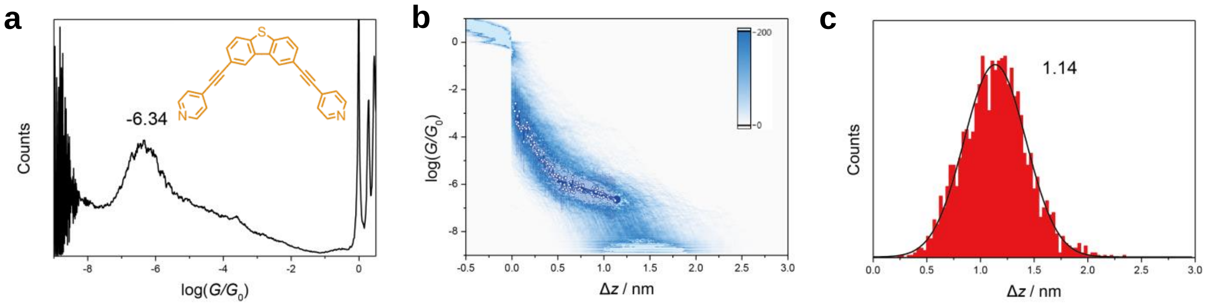


**Supplementary Figure 29.** (**a**) 1D conductance histograms, (**b**) 2D histogram and (**c**) characteristic plateau length distribution between $10^{-0.30}$ $G_0$ and $10^{-7.28}$ $G_0$ in MCBJ experiment of molecule **6-S*m***. In the 2D histogram, statistically averaged conductance−distance traces (hollow circles) with variations indicated by the standard deviations (bars) are shown, along with the linear fit (line). The solid circle represents the last data point in the linear fit before junction rupture.

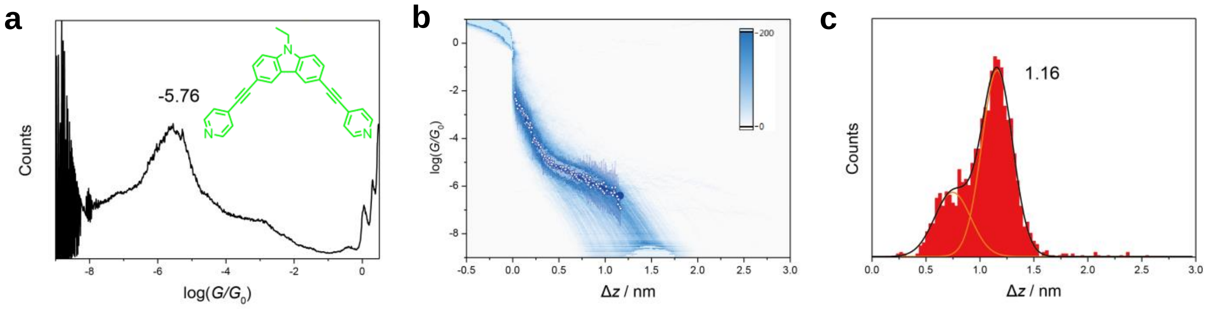


**Supplementary Figure 30.** (**a**) 1D conductance histograms, (**b**) 2D histogram and (**c**) characteristic plateau length distribution between $10^{-0.30}$ $G_0$ and $10^{-6.93}$ $G_0$ in MCBJ experiment of molecule **7-N*m***. In the 2D histogram, statistically averaged conductance−distance traces (hollow circles) with variations indicated by the standard deviations (bars) are shown, along with the linear fit (line). The solid circle represents the last data point in the linear fit before junction rupture.

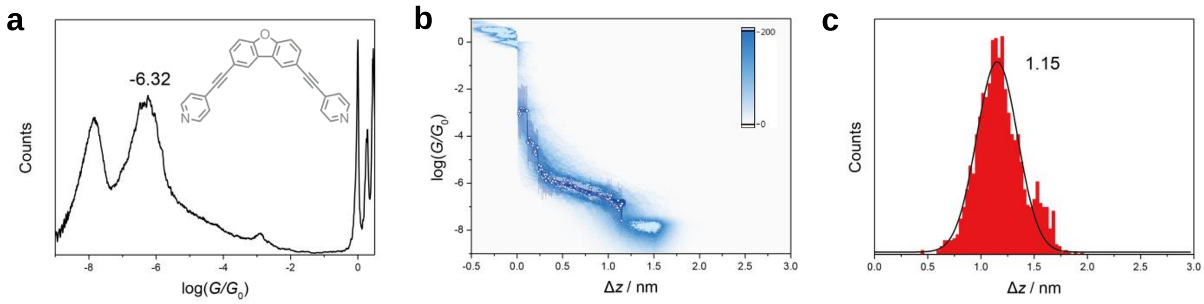


**Supplementary Figure 31.** (**a**) 1D conductance histogram, (**b**) 2D histogram and (**c**) characteristic plateau length distribution between $10^{-0.30}$ $G_0$ and $10^{-7.30}$ $G_0$ in MCBJ experiment of molecule **8-O*m***. In the 2D histogram, statistically averaged conductance−distance traces (hollow circles) with variations indicated by the standard deviations (bars) are shown, along with the linear fit (line). The solid circle represents the last data point in the linear fit before junction rupture.

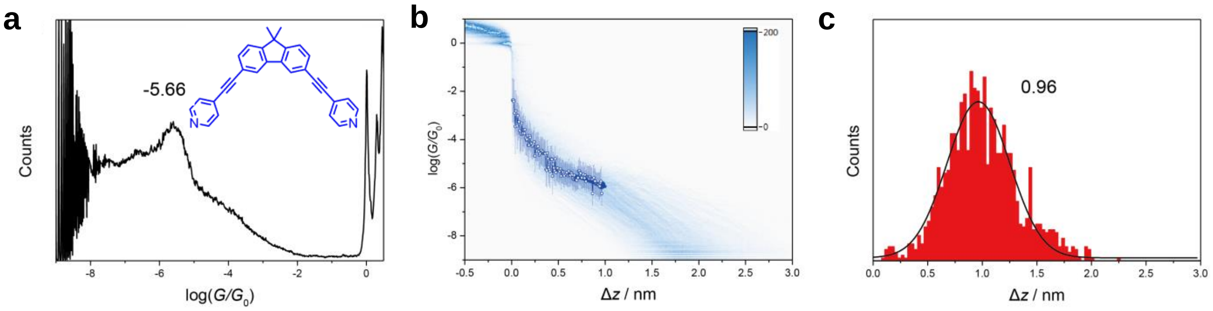


**Supplementary Figure 32.** (**a**) 1D conductance histogram, (**b**) 2D histogram and (**c**) characteristic plateau length distribution between $10^{-0.30}$ $G_0$ and $10^{-6.16}$ $G_0$ in MCBJ experiment of molecule **9-C*m***. In the 2D histogram, statistically averaged conductance–distance traces (hollow circles) with variations indicated by the standard deviations (bars) are shown, along with the linear fit (line). The solid circle represents the last data point in the linear fit before junction rupture.

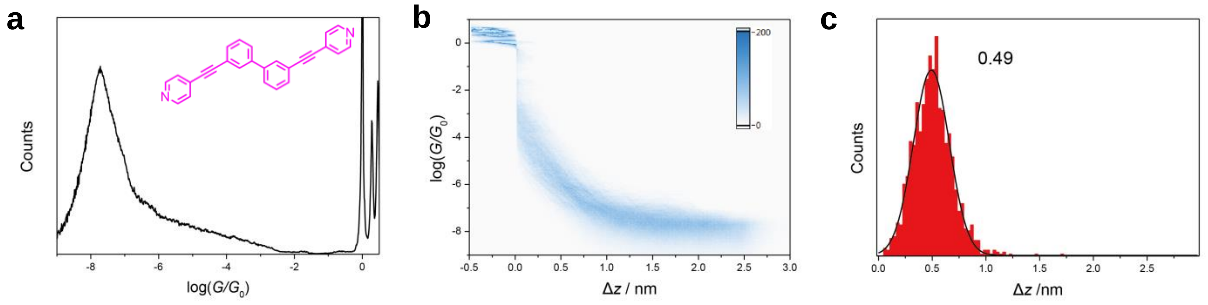


**Supplementary Figure 33.** (**a**) 1D conductance histogram, (**b**) 2D histogram and (**c**) characteristic plateau length distribution between $10^{-0.30}$ $G_0$ and $10^{-6.00}$ $G_0$ in MCBJ experiment of molecule **10-2H*m***. In this case, no discernible conductance plateaus can be observed and the peak in (c) should be assigned to pure tunnelling.

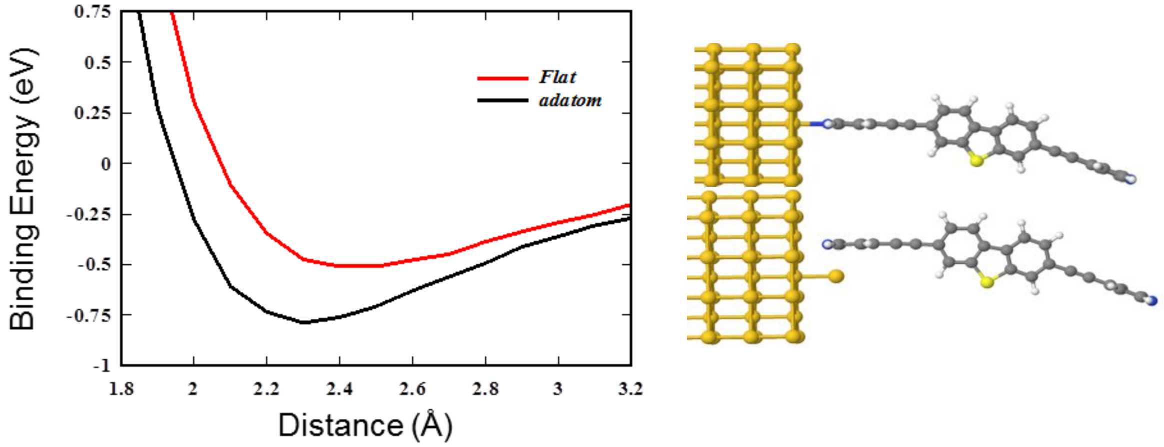


**Supplementary Figure 34.** Binding energies as a function of the distance (Å) of the nitrogen from the electrode surface (left). Examples of binding geometries on a flat electrode and an electrode containing an adatom (right).

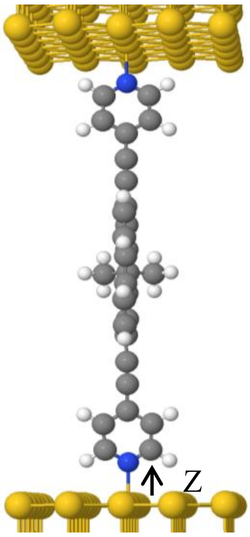


**Supplementary Figure 35.** A sketch showing the distance z of the nitrogen from the gold surface. The equilibrium distance is found to be approximately 2.3 Å from the minimization of the binding energy.

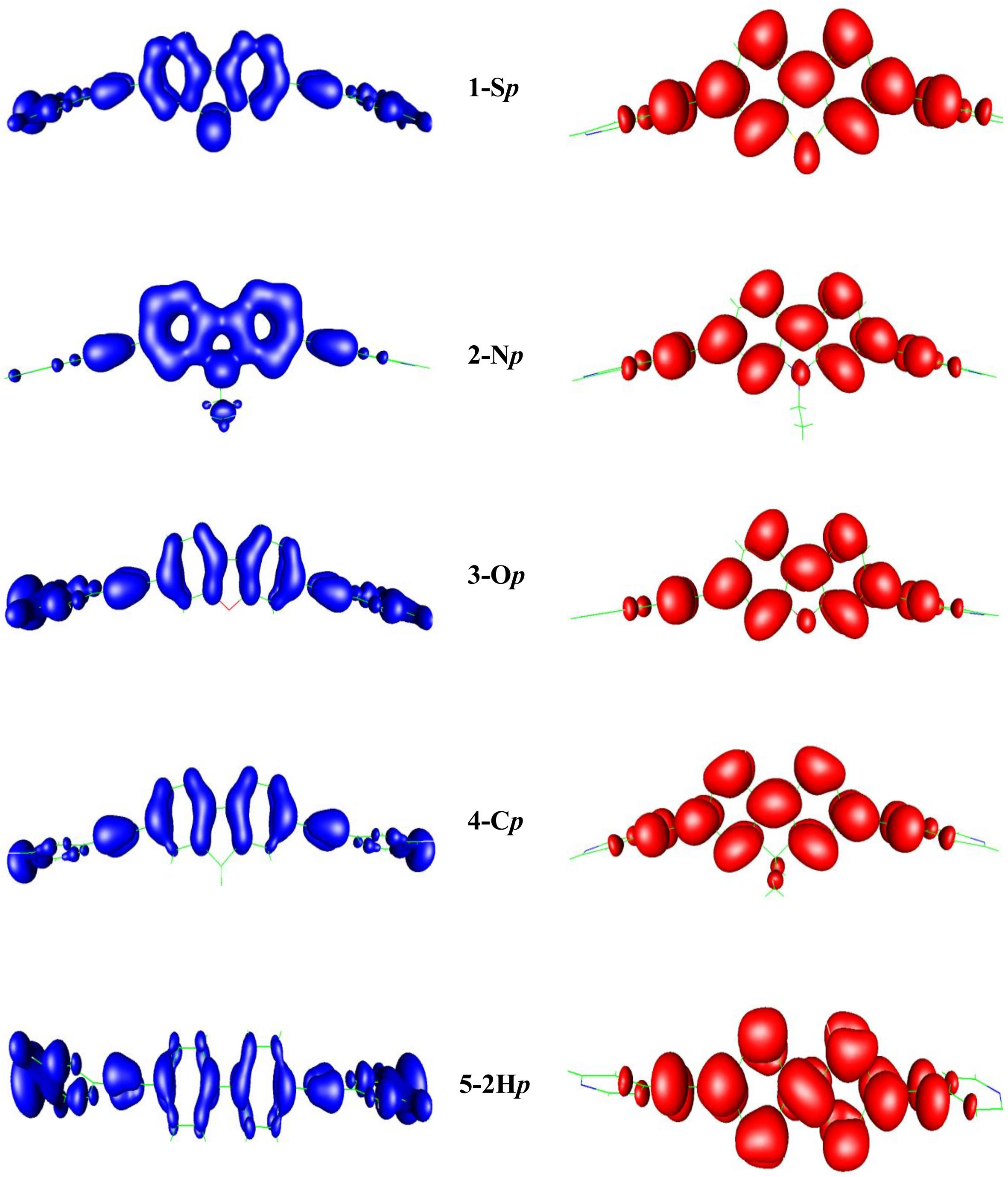


**Supplementary Figure 36.** Gas phase calculations of local density of states of the HOMO (blue) and LUMO orbitals (red) for *para*-linked molecules.

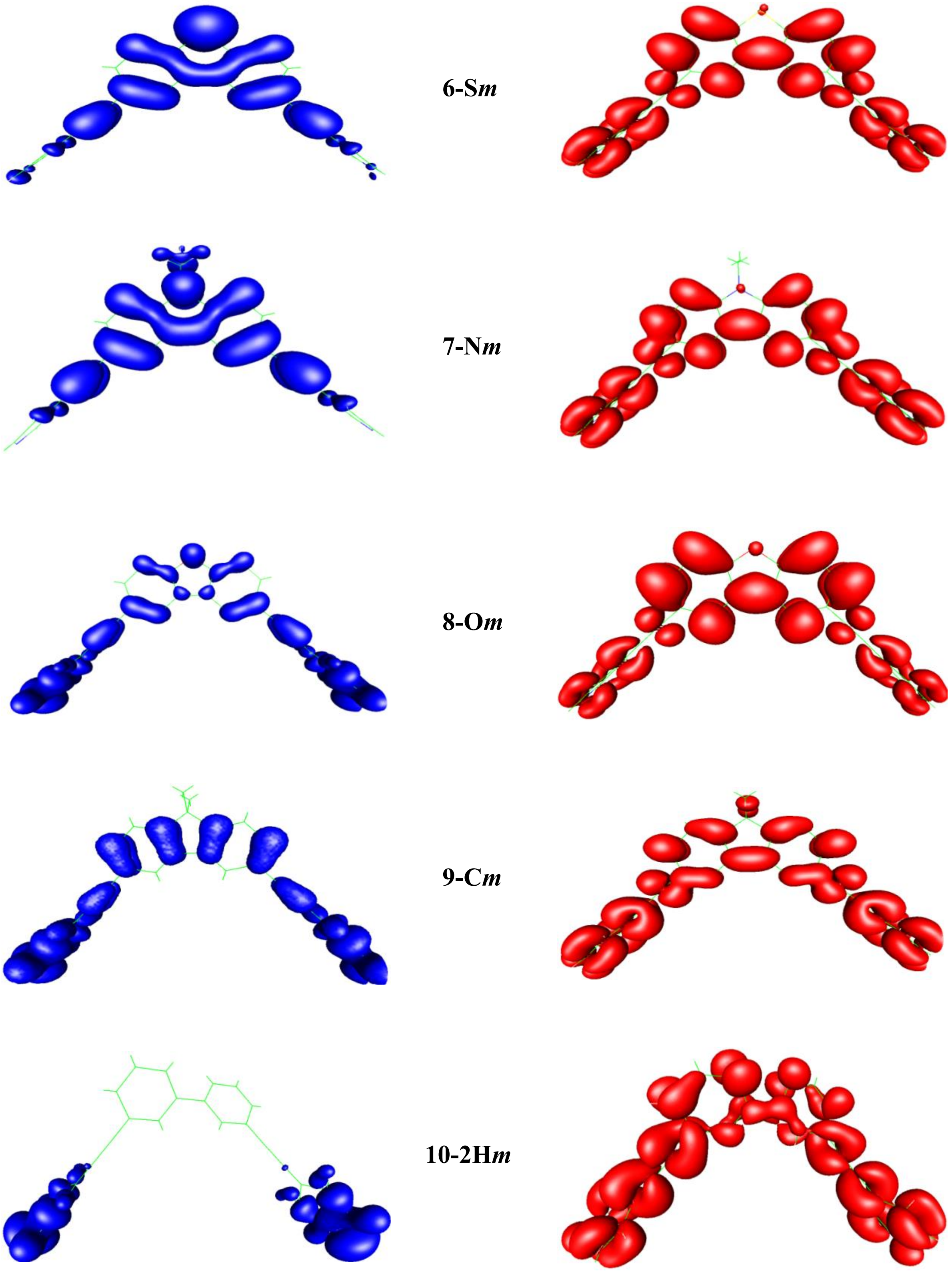


**Supplementary Figure 37.** Gas phase calculations of local density of states of the HOMO (blue) and LUMO orbitals (red) for *meta*-linked molecules.

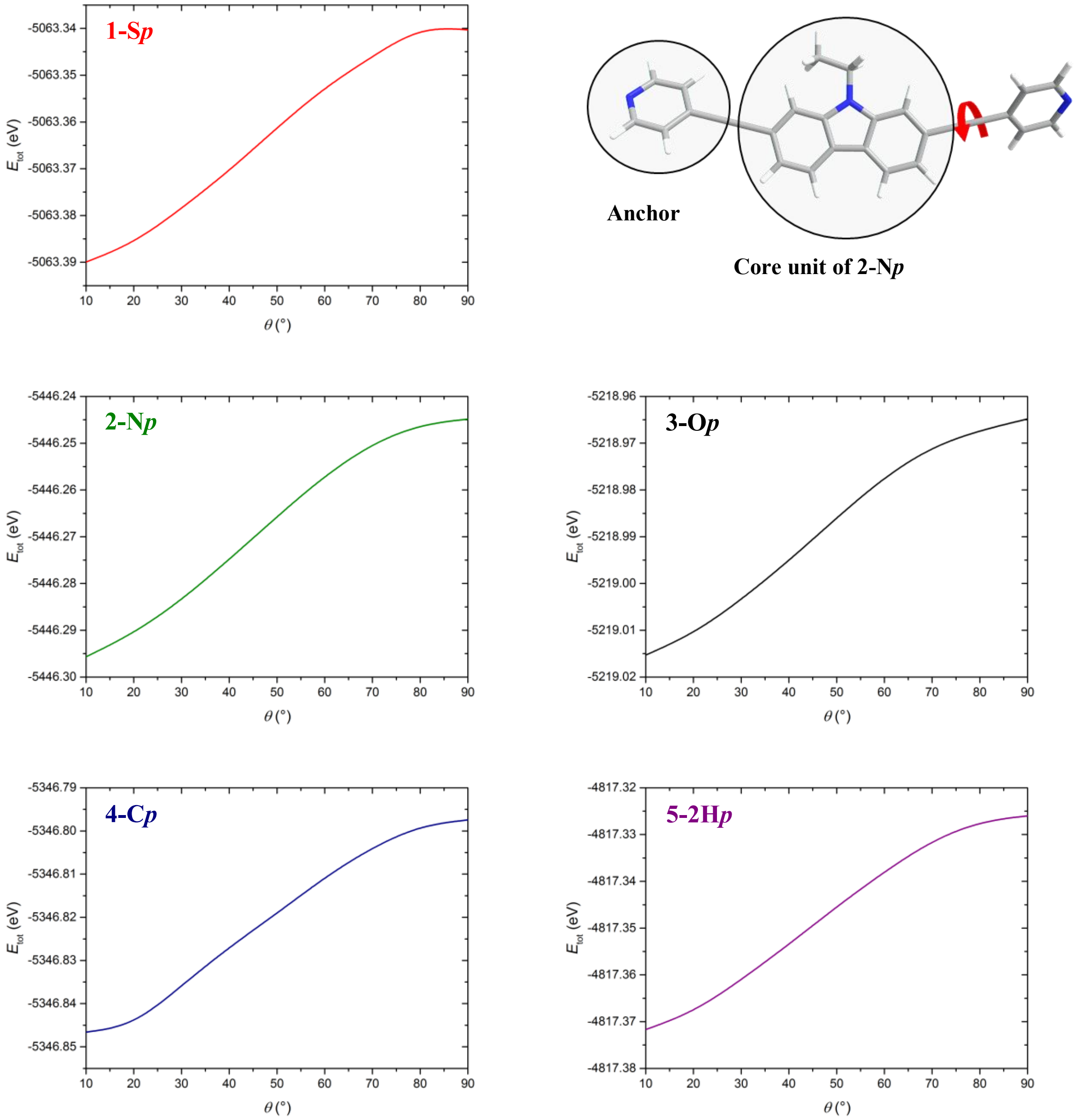


**Supplementary Figure 38.** Total energy versus angle rotation of anchoring groups with respect to the central unit of *para*-linked molecules, to find the correct angle between the anchoring group and the core of the molecule.

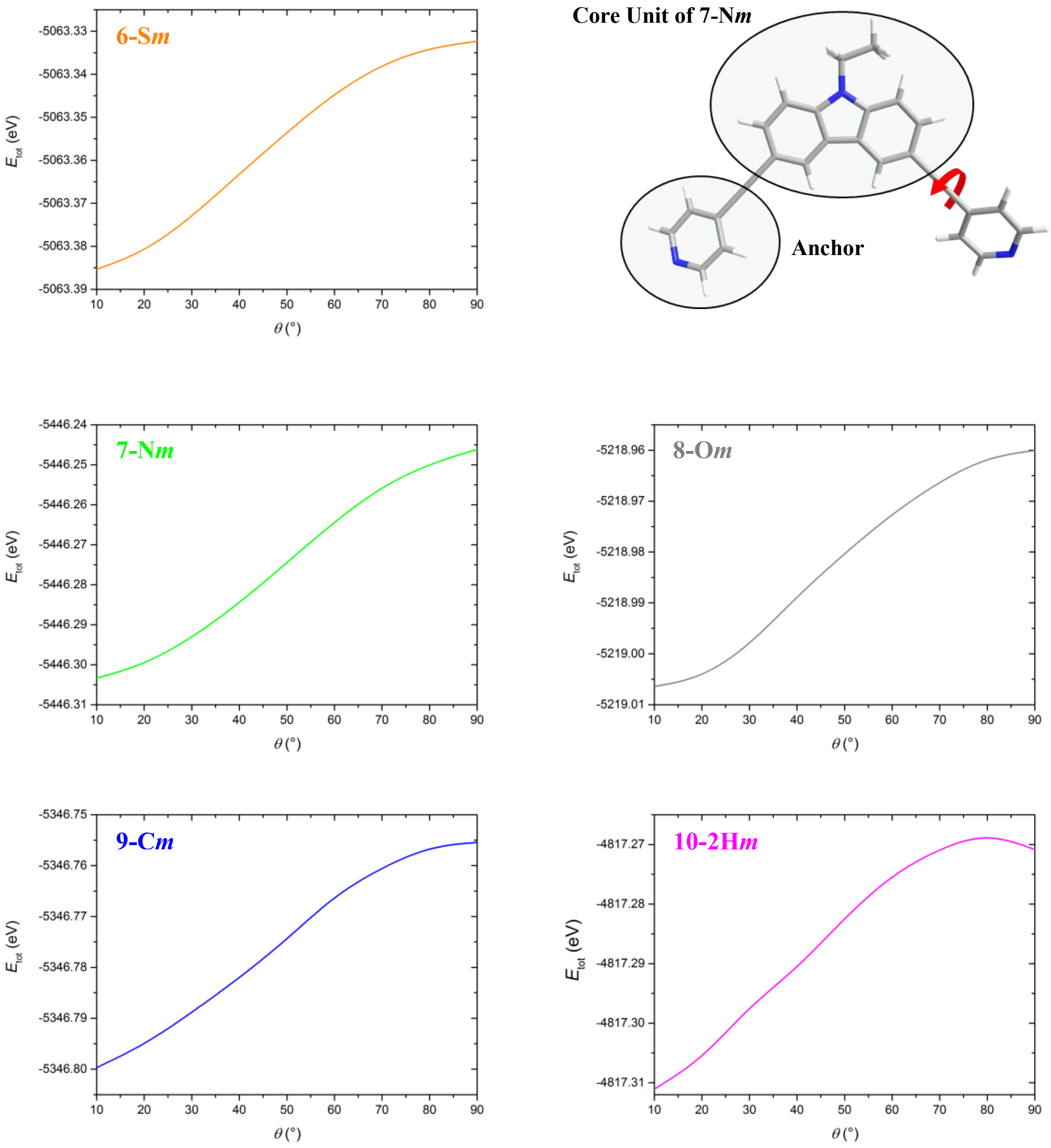


**Supplementary Figure 39.** Total energy versus angle rotation of anchoring groups with respect to the central unit of *meta*-linked molecules, to find the correct angle between the anchoring group and the core of the molecule.

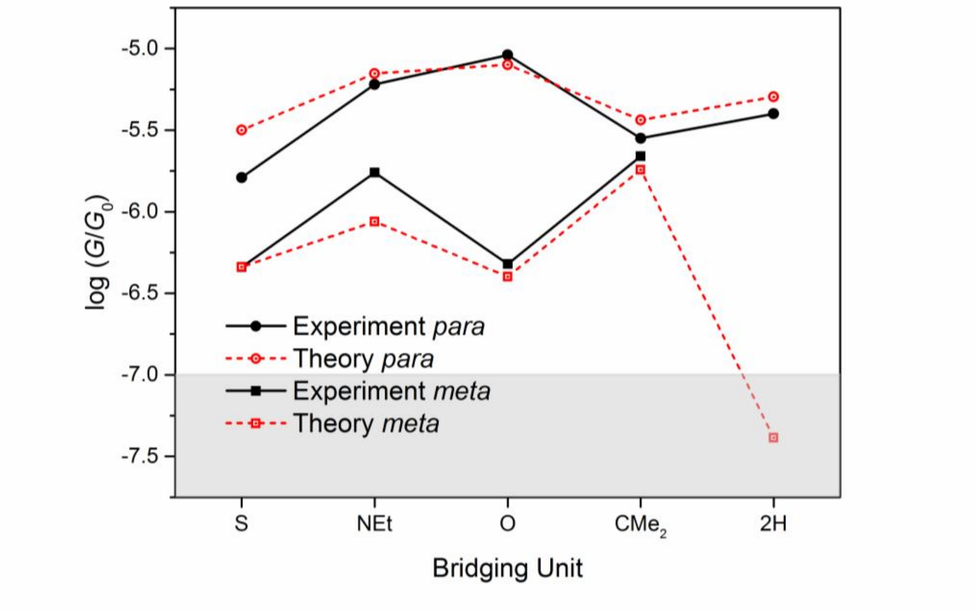


**Supplementary Figure 40.** Comparison between theoretical and experimental result for most probable conductance. The grey area indicates the detection limit of the MCBJ-setup.

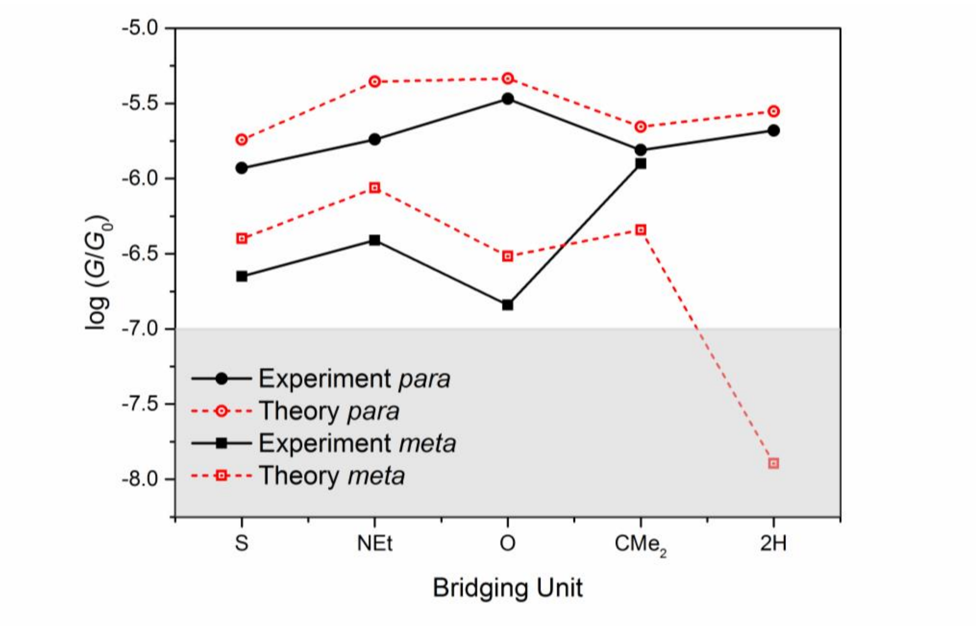


**Supplementary Figure 41.** Comparison between theoretical and experimental result for fully stretched conductance. The grey area indicates the detection limit of the MCBJ-setup.

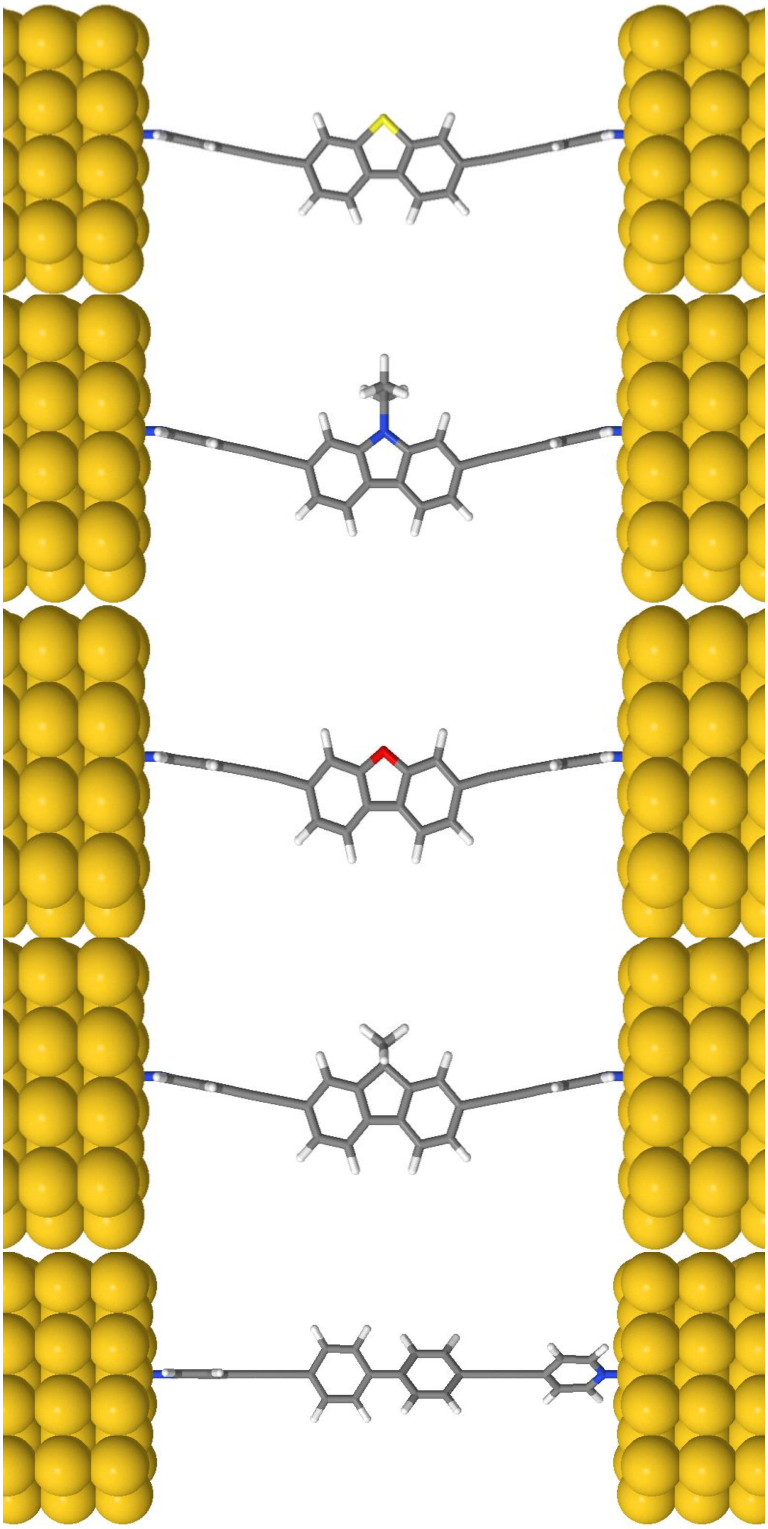

**Supplementary Figure 42.** Geometry of the molecular junction containing *para*-linked molecular wires **1–5**.

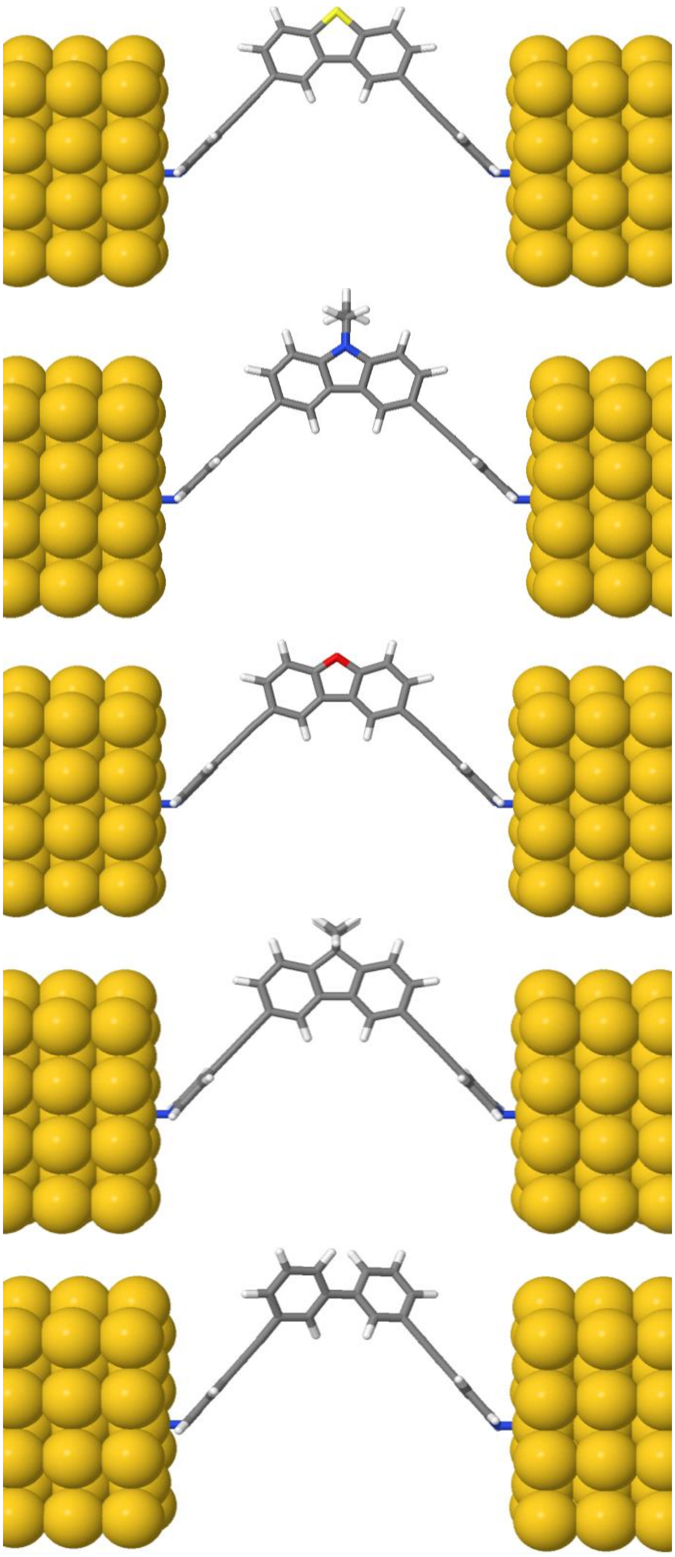

**Supplementary Figure 43.** Geometry of the molecular junction containing *meta*-linked molecular wires **6–10**.

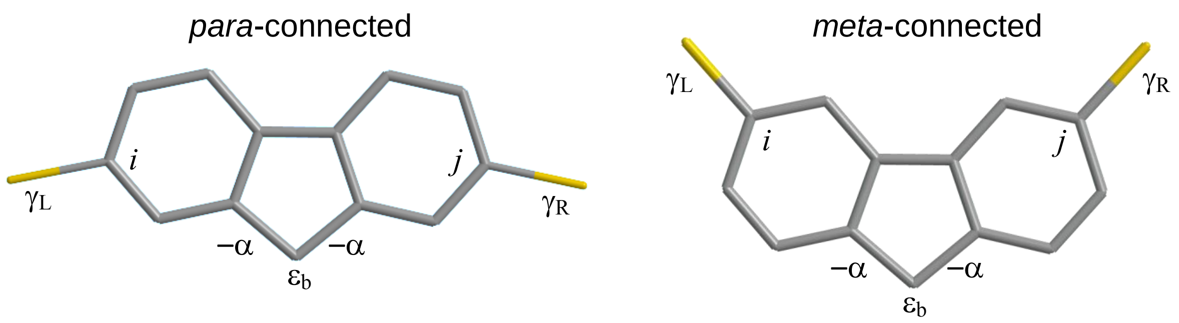


**Supplementary Figure 44.** Tight-binding models of *para*- and *meta*-connected molecules. Within the core, all site energies are zero, except that marked $\varepsilon_b$. For nearest neighbour sites *i*,*j* the nearest neighbour Hamiltonian matrix elements $H_{ij}$ are equal to $-\gamma$, where $\gamma = 1$, except for those matrix elements denoted $-\alpha$, which connect to the pendant site and $-\gamma_L = -\gamma_R = -0.08$, which connect the core sites *i* and *j* to external electrodes. The electrodes are simple 1-dimensional tight binding chains formed from single-orbital atoms with site energies of zero and nearest neighbour coupling –1. The dispersion relation for such a chain is $E = -2\cos k$ and therefore the wave-vector of an electron of energy $E$ in such a chain is $k(E) = \text{acos}(-E/2)$.

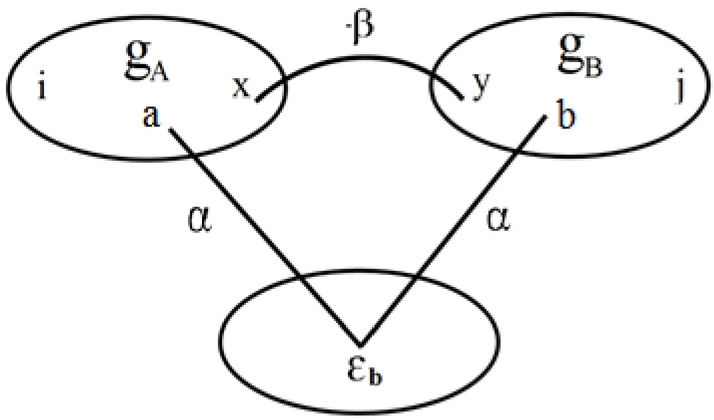


**Supplementary Figure 45.** An abstraction of the isolated core.

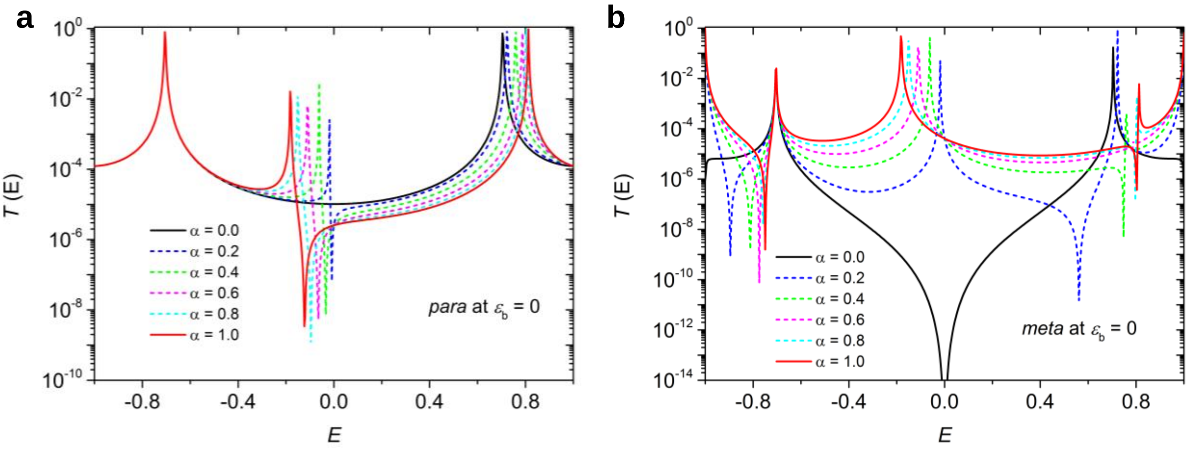


**Supplementary Figure 46.** ***T*** **(E) vs.** ***E*** **for different values of** $\boldsymbol{\alpha}$ **at** $\boldsymbol{\gamma_b}$ **= 0.** Transmission coefficients *T* (E) for *para*-connection (**a**) and *meta*-connection (**b**), when $\varepsilon_b = 0$.

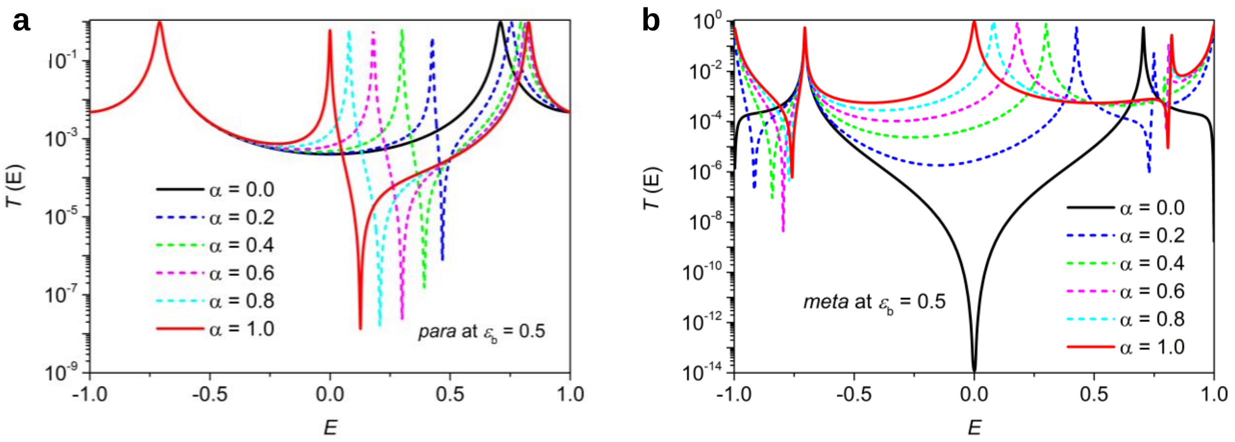


**Supplementary Figure 47.** ***T*** **(E) vs.** ***E*** **for different values of** $\boldsymbol{\alpha}$ **at** $\boldsymbol{\gamma_b}$ **= 0.5.** Transmission coefficients *T* (E) for *para*-connection (**a**) and *meta*-connection (**b**), when $\varepsilon_b = 0.5$.

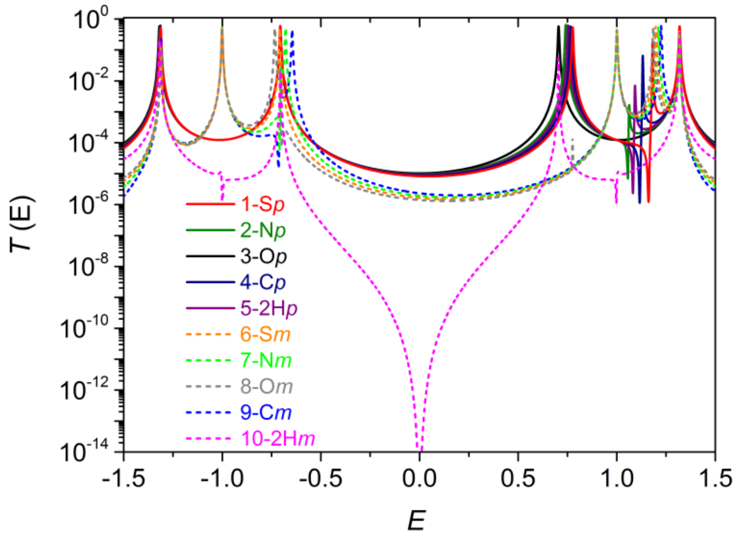


**Supplementary Figure 48.** Tight binding results obtained by tuning the values of $\varepsilon_b$, where $\alpha = 1$, $\gamma_L = \gamma_R = 0.08$.

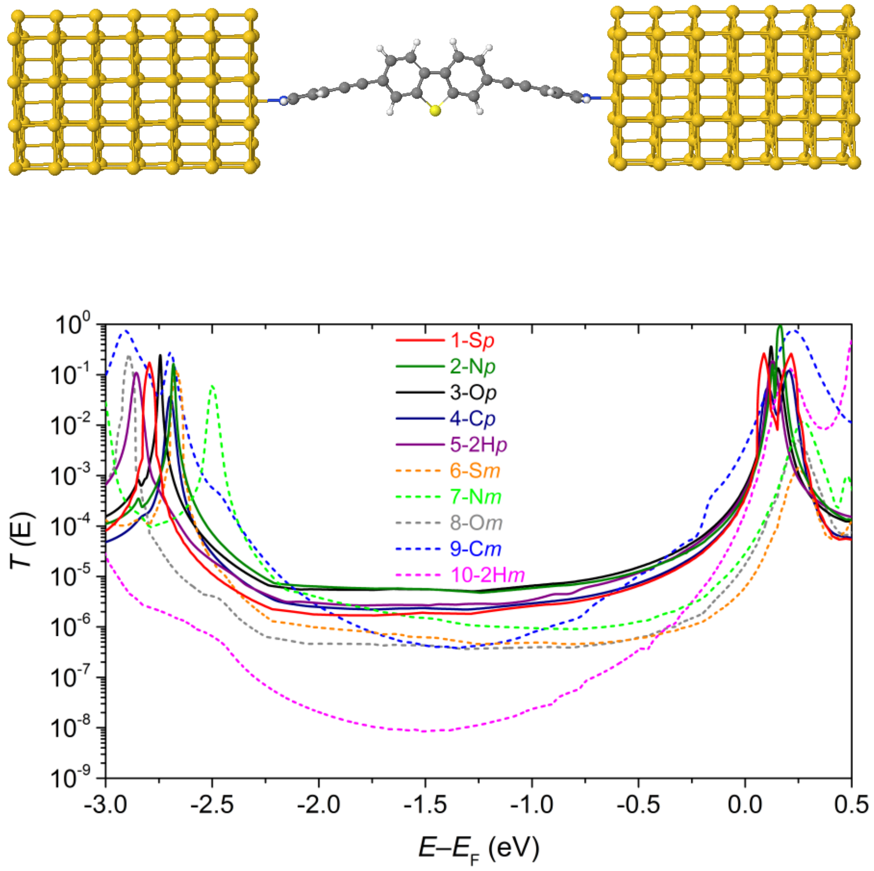


**Supplementary Figure 49.** Example of binding geometry of a molecule located between flat electrodes (top). DFT transmission functions for *para*- and *meta*-linked compounds **1–10** (bottom)**.** It is known that the DFT-predicted Fermi energy ($E_F$ = 0.0 eV in Supplementary Figure 49) is usually unreliable and therefore to obtain agreement with experiment, we adjusted this single parameter to obtain the closest fit to the 10 experimentally measured conductances. This yields a value of $E_F$ = –0.8 eV. With this choice, the predicted room-temperature conductances are shown in Supplementary Figure 40 and Supplementary Figure 41.

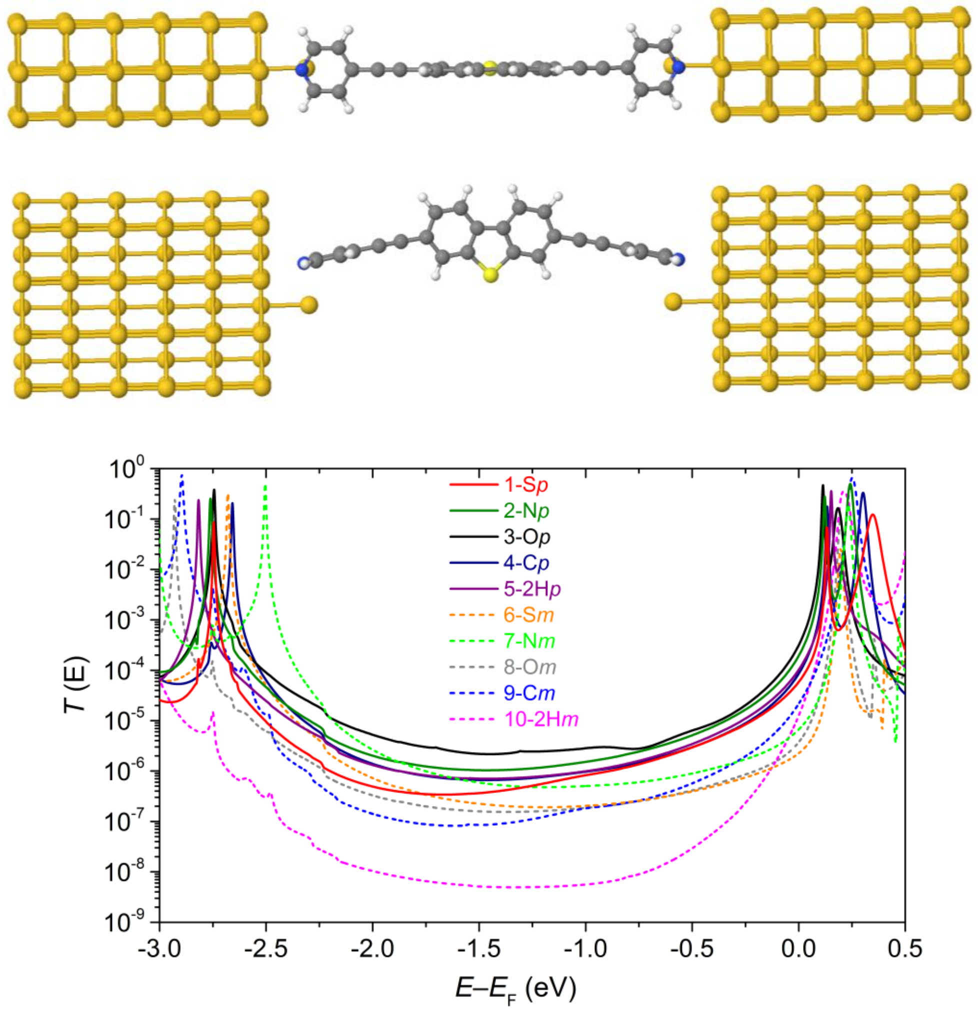


**Supplementary Figure 50.** Examples of binding geometry of a molecule located between flat electrodes containing adatoms (top). DFT transmission functions for *para*- and *meta*-linked compounds **1–10** (bottom).

# Supplementary Tables

**Supplementary Table 1.** Photophysical data of compounds **1–10**.

| Compound *para*-series | $\lambda_{max\ UV}$ (nm)[a] | *e* ($M^{-1}\ cm^{-1}$) | $\lambda_{onset\ UV}$ (nm)[a] | $E_g$ (eV)[b] | Compound *meta*-series | $\lambda_{max\ UV}$ (nm)[a] | $\varepsilon$ ($M^{-1}\ cm^{-1}$) | $\lambda_{onset\ UV}$ (nm)[a] | $E_g$ (eV)[b] |
|---|---|---|---|---|---|---|---|---|---|
| **1-S*p*** | 339 | 74950 | 381 | 3.25 | **6-S*m*** | 307 | 56434 | 359 | 3.45 |
| | 299 | 24631 | | | | 270 | 63279 | | |
| | 257 | 39916 | | | | | | | |
| **2-N*p*** | 352 | 79046 | 414 | 2.99 | **7-N*m*** | 355 | 52299 | 385 | 3.22 |
| | 292 | 25212 | | | | 312 | 65276 | | |
| | 265 | 55818 | | | | 248 | 42964 | | |
| **3-O*p*** | 349 | 74864 | 387 | 3.20 | **8-O*m*** | 314 | 50599 | 338 | 3.67 |
| | 288 | 21046 | | | | 305 | 54844 | | |
| | | | | | | 260 | 45130 | | |
| **4-C*p*** | 352 | 72251 | 385 | 3.22 | **9-C*m*** | 330 | 21158 | 348 | 3.56 |
| | 316 | 33021 | | | | 316 | 24588 | | |
| | 304 | 26571 | | | | 276 | 76717 | | |
| **5-2H*p*** | 327 | 73911 | 375 | 3.31 | **10-2H*m*** | 301 | 32887 | 321 | 3.86 |
| | | | | | | 283 | 39104 | | |
| | | | | | | 267 | 35398 | | |

[a]Data are obtained from the UV–Vis absorption measurements in dilute dichloromethane solution at room temperature. [b]$E_g = 1240/\lambda_{onset\ UV.}$

**Supplementary Table 2.** Experimental measurements and theoretical calculations of HOMO–LUMO band gaps.

| Compound *para*-series | $E_g$ (eV)[a] | $E_{g\ DFT}$ iso. (eV)[b] | $E_{g\ DFT}$ M····Au (eV)[c] | Compound *meta*-series | $E_g$ (eV)[a] | $E_{g\ DFT}$ iso. (eV)[b] | $E_{g\ DFT}$ M····Au (eV)[c] |
|---|---|---|---|---|---|---|---|
| **1-S*p*** | 3.25 | 2.87 | 2.71 | **6-S*m*** | 3.45 | 3.07 | 2.46 |
| **2-N*p*** | 2.99 | 2.83 | 2.60 | **7-N*m*** | 3.22 | 2.96 | 2.30 |
| **3-O*p*** | 3.20 | 2.84 | 2.62 | **8-O*m*** | 3.67 | 3.24 | 2.68 |
| **4-C*p*** | 3.22 | 2.78 | 2.61 | **9-C*m*** | 3.56 | 3.25 | 2.52 |
| **5-2H*p*** | 3.31 | 2.96 | 2.73 | **10-2H*m*** | 3.86 | 3.23 | 3.01 |

[a] $E_g = 1240/\lambda_{onset\ UV.}$ [b]Theoretical HOMO–LUMO band gaps for isolated molecule. [c]Theoretical HOMO–LUMO band gaps for molecule anchored to gold clusters.

**Supplementary Table 3.** Experimental measurements and theoretical calculations ($E_F$ = –0.8 eV) at the most probable conductance for both *para* and *meta*-linked compounds **1–10**.

| Bridging Unit | Experimental conductance *para* (log($G/G_0$)) | Theoretical conductance *para* (log($G/G_0$)) | Experimental conductance *meta* (log($G/G_0$)) | Theoretical conductance *meta* (log($G/G_0$)) |
|---|---|---|---|---|
| S | –5.79 ± 0.30 | –5.50 | –6.34 ± 0.47 | –6.34 |
| NEt | –5.22 ± 0.40 | –5.15 | –5.76 ± 0.48 | –6.06 |
| O | –5.04 ± 0.47 | –5.10 | –6.32 ± 0.38 | –6.40 |
| $CMe_2$ | –5.55 ± 0.44 | –5.44 | –5.66 ± 0.82 | –5.74 |
| 2H | –5.40 ± 0.36 | –5.30 | < –7 | –7.38 |

**Supplementary Table 4.** Experimental measurements and theoretical calculation ($E_F$ = –1.2) at fully stretched conductance for both *para* and *meta*-linked compounds **1–10**.

| Bridging Unit | Experimental conductance *para* (log($G/G_0$)) | Theoretical conductance *para* (log($G/G_0$)) | Experimental conductance *meta* (log($G/G_0$)) | Theoretical conductance *meta* (log($G/G_0$)) |
|---|---|---|---|---|
| S | –5.93 ± 0.16 | –5.74 | –6.65 ± 0.35 | –6.40 |
| NEt | –5.74 ± 0.17 | –5.36 | –6.41 ± 0.25 | –6.06 |
| O | –5.47 ± 0.22 | –5.33 | –6.84 ± 0.24 | –6.52 |
| $CMe_2$ | –5.81 ± 0.12 | –5.66 | –5.90 ± 0.39 | –6.34 |
| 2H | –5.68 ± 0.11 | –5.55 | < –7 | –7.89 |

**Supplementary Table 5.** Comparison between experimental conductances (column 2) and theoretical values. Columns 3-6 show DFT-GOLLUM results obtained at different Fermi energies (relative to the DFT-predicted Fermi energy). Column 7 shows the conductance obtained from the tight binding model by choosing values of $\varepsilon_b$, which yield closest agreement with experiment. Where $\alpha = -1$, $\gamma_L = \gamma_R = -0.08$

| Compound | MCBJ (log(*G*/*G*$_0$)) | DFT (log(*G*/*G*$_0$)) | | | | Tight-binding conductance | $\varepsilon_b$ | Mulliken charge | Electronegativity[1] |
|---|---|---|---|---|---|---|---|---|---|
| | | $E_F = -0.5$ | $E_F = -0.8$ | $E_F = -1.2$ | $E_F = -1.5$ | | | | |
| **1-S*p*** | –5.79 | –5.18 | –5.50 | –5.74 | –5.73 | –5.06 | –1.8 | 6.04 | 2.58 |
| **2-N*p*** | –5.22 | –4.89 | –5.15 | –5.33 | –5.26 | –5.04 | –2.0 | 4.50 | 3.04 |
| **3-O*p*** | –5.04 | –4.81 | –5.10 | –5.31 | –5.27 | –5.02 | –2.2 | 5.73 | 3.44 |
| **4-C*p*** | –5.55 | –5.12 | –5.44 | –5.66 | –5.64 | –5.08 | –1.6 | 3.83 | 2.55 |
| **5-2H*p*** | –5.40 | –4.92 | –5.30 | –5.55 | –5.58 | –4.99 | | α = 0 | |
| **6-S*m*** | –6.34 | –6.23 | –6.33 | –6.39 | –6.18 | –5.71 | –1.8 | 6.04 | 2.58 |
| **7-N*m*** | –5.76 | –5.89 | –6.06 | –6.06 | –5.81 | –5.79 | –2.0 | 4.50 | 3.04 |
| **8-O*m*** | –6.32 | –6.21 | –6.40 | –6.48 | –6.36 | –5.85 | –2.2 | 5.72 | 3.44 |
| **9-C*m*** | –5.66 | –4.97 | –5.74 | –6.34 | –6.36 | –5.63 | –1.6 | 3.83 | 2.55 |
| **10-2H*m*** | < –7 | –6.46 | –7.28 | –7.89 | –8.09 | – | | α = 0 | |

# Supplementary Notes

## Supplementary Note 1. Synthesis and Characterization of Molecules.

### General Information

All reactions were carried out under an argon atmosphere unless otherwise stated. Starting materials were purchased commercially and were used as received. Solvents were dried using an Innovative Technology solvent purification system and were stored in ampoules under argon. Analytical thin layer chromatography (TLC) was performed on 20 mm pre-coated plates of silica gel (Merck, silica gel 60 $F_{254}$) TLC plates and spots were visualised using a TLC lamp emitting at 365, 312 or 254 nm. Silica gel column chromatography was performed using silica gel 60 purchased from Sigma Aldrich. UV–Visible absorption spectra were recorded at room temperature on a Thermo Scientific Evolution 220 spectrometer. $^{1}$H and $^{13}$C NMR spectroscopy was carried out on Bruker AV400, Varian VNMRS 500 and 700, and Varian Inova 500 NMR spectrometers. Chemical shifts are reported in ppm relative to $CHCl_3$ (7.26 ppm), and all NMR data was processed in MestReNova V10. Melting points were carried out on a Stuart SMP40 machine using open ended capillaries with a ramping rate of 1 °C min$^{-1}$. Videos were replayed manually to determine the melting point. High resolution mass spectroscopy was carried out on a Waters LCT Premier XE using ASAP ionisation. Samples were analysed directly as solids. Elemental analysis was performed on an Exeter Analytical E-440 machine.

### General Procedures for Sonogashira Cross-Coupling

A solution of 4-((trimethylsilyl)ethynyl)pyridine (**24**) (4.0 equivalents) in dry 1,4-dioxane (5.2 mL per mmol) was treated with tetrabutylammonium fluoride (TBAF) in THF (1.0 mL per mmol, 1 M) and stirred in darkness for 30 min at room temperature. This solution was cannulated into an oven-dried flask charged with dibromide, $PdCl_2(PhCN)_2$ (10% mmol), CuI (6.0% mmol), *t*-$Bu_3P$ (15% mmol), and diisopropylamine ((*i*-Pr)$_2$NH, 9.6 mL per mmol). The reaction mixture was stirred at 85 ºC until TLC showed full conversion of the starting material. After the reaction was complete, the mixture was diluted with DCM (20 mL per mmol) filtered through a pad of Celite™, washed with saturated aqueous $NH_4Cl$ and NaCl solutions, and dried with $MgSO_4$. Solvents were removed at reduced pressure and the crude product was further purified by flash column chromatography.

## Synthesis of *para*-Series

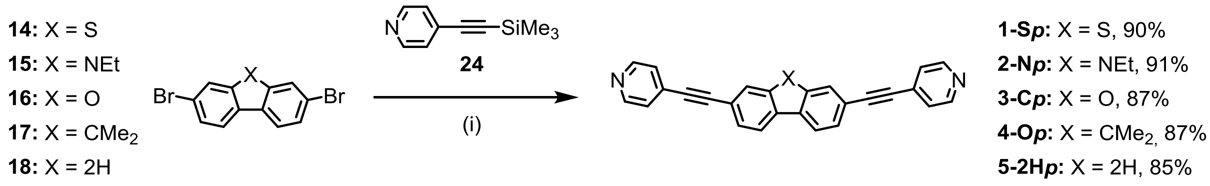


**Scheme 1.** Synthesis of *para* conjugated compounds **1–5**. Reagents and Conditions: (i) 4-((trimethylsilyl)ethynyl)pyridine **24**, TBAF (1 M in THF), 1,4-dioxane, rt, 30 min; then $PdCl_2(PhCN)_2$, CuI, *t*-$Bu_3P$, (*i*-$Pr)_2NH$, 85 °C, 3–12 h.

### 3,7-bis(pyridin-4-ylethynyl)dibenzo[b,d]thiophene (1-S*p*)

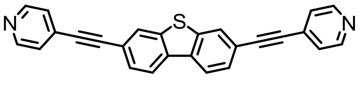


*General Procedure*: **24** (1.0 g, 5.7 mmol) in 1,4-dioxane (30 mL), TBAF (1 M in THF, 5.7 mL), 30 min, rt. Cannulated into flask charged with dibromide **14**[2] (0.50 g, 1.46 mmol), $PdCl_2(PhCN)_2$ (56 mg), CuI (17 mg), *t*-$Bu_3P$ (44 mg), and (*i*-$Pr)_2NH$ (14 mL), 12 h, 85 ºC. Crude product was purified by flash column chromatography ($SiO_2$, toluene/ethyl acetate, 5:1 (v/v), 5% triethylamine) affording **1-S*p*** (0.51 g, 1.3 mmol, 90%) as a yellow solid. mp: 272.2–272.9 °C. $^1$H NMR (400 MHz, $CDCl_3$, δ): 8.64–8.63 (m, 4H), 8.15 (dd, *J* = 8.2, 0.7 Hz, 2H), 8.07 (dd, *J* = 1.4, 0.7 Hz, 2H), 7.65 (dd, *J* = 8.2, 1.4 Hz, 2H), 7.43–7.40 (m, 4H). $^{13}$C NMR (101 MHz, $CDCl_3$, δ): 149.91, 140.33, 135.53, 131.43, 128.41, 126.52, 125.69, 122.05, 121.08, 93.97, 87.97. HRMS–ASAP$^+$ (m/z): calcd for $C_{26}H_{14}N_2S$ $[M]^{•+}$, 386.0878; found, 386.0878. Anal. calcd for $C_{26}H_{14}N_2S$: C 80.80; H 3.65; N 7.25; found: C 80.66; H 3.57; N 7.21.

**9-ethyl-2,7-bis(pyridin-4-ylethynyl)-9H-carbazole (2-N*p*)**

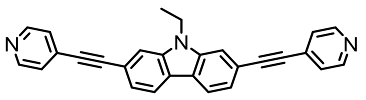

*General Procedure*: **24** (1.0 g, 5.7 mmol) in 1,4-dioxane (30 mL), TBAF (1 M in THF, 5.7 mL), 30 min, rt. Cannulated into flask charged with dibromide **15**[3] (0.50 g, 1.4 mmol), $PdCl_2(PhCN)_2$ (54 mg), CuI (16 mg), *t*-$Bu_3P$ (43 mg), and (*i*-Pr)$_2$NH (13 mL), 6 h, 85 ºC. Crude product was purified by flash column chromatography ($SiO_2$, toluene/ethyl acetate, 5:1 (v/v), 5% triethylamine) affording **2-N*p*** (0.51 g, 1.3 mmol, 91%) as a yellow solid. mp: 276.2–277.2 °C. $^1$H NMR (400 MHz, $CDCl_3$, δ): 8.64–8.63 (m, 4H), 8.09 (dd, *J* = 8.1, 0.7 Hz, 2H), 7.65 (dd, *J* = 1.3, 0.7 Hz, 2H), 7.45 (dd, *J* = 8.1, 1.3 Hz, 2H), 7.46–7.44 (m, 4H), 4.41 (q, *J* = 7.2, 2H), 1.50 (t, *J* = 7.2, 3H). $^{13}$C NMR (101 MHz, $CDCl_3$, δ): 149.92, 140.36, 131.70, 125.64, 123.36, 123.32, 120.97, 119.67, 112.42, 95.43, 86.92, 37.92, 14.04. HRMS–ASAP$^+$ (m/z): calcd for $C_{28}H_{19}N_3$ $[M]^{\bullet+}$, 397.1579; found, 397.1581. Anal. calcd for $C_{28}H_{19}N_3$: C 84.61; H 4.82; N 10.57; found: C 84.35; H 4.79; N 10.55.

**3,7-bis(pyridin-4-ylethynyl)dibenzo[b,d]furan (3-O*p*)**

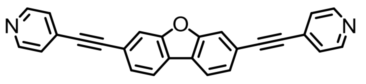

*General Procedure*: **24** (0.26 g, 1.23 mmol) in 1,4-dioxane (6.4 mL), TBAF (1 M in THF, 1.23 mL), 30 min, rt. Cannulated into flask charged with dibromide **16**[4] (0.10 g, 0.31 mmol), $PdCl_2(PhCN)_2$ (12 mg), CuI (3.5 mg), *t*-$Bu_3P$ (9.3 mg), and (*i*-Pr)$_2$NH (2.8 mL), 5 h, 85 ºC. Crude product was purified by flash column chromatography ($SiO_2$, toluene/ethyl acetate, 5:1 (v/v), 5% triethylamine) affording **3-O*p*** (0.10 g, 0.27 mmol, 87%) as a white solid. mp: 226.8–227.4 °C. $^1$H NMR (400 MHz, $CDCl_3$, δ): 8.65–8.63 (m, 4H), 7.96 (dd, *J* = 8.1, 0.6 Hz, 2H), 7.78 (dd, *J* = 1.3, 0.6 Hz, 2H), 7.58 (dd, *J* = 8.1, 1.3 Hz, 2H), 7.46–7.44 (m, 4H). $^{13}$C NMR (101 MHz, $CDCl_3$, δ): 156.51, 149.62, 131.72, 127.35, 125.79, 124.86, 121.47, 121.17, 115.40, 94.43, 87.77. HRMS–ASAP$^+$ (m/z): calcd for $C_{26}H_{15}N_2O$ $[M+H]^+$, 371.1184; found, 371.1178. Anal. calcd for $C_{26}H_{14}N_2O$: C 84.31; H 3.81; N 7.56; found: C 83.89; H 3.76; N 7.55.

**4,4'-((9,9-dimethyl-9H-fluorene-2,7-diyl)bis(ethyne-2,1-diyl))dipyridine (4-C*p*)**

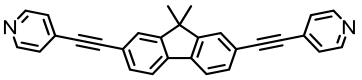

*General Procedure*: **24** (0.80 g, 4.5 mmol) in 1,4-dioxane (24 mL), TBAF (1 M in THF, 4.5 mL), 30 min, rt. Cannulated into flask charged with dibromide **17**[5] (0.40 g, 1.1 mmol), $PdCl_2(PhCN)_2$ (44 mg), CuI (13 mg), *t*-$Bu_3P$ (34 mg), and (*i*-$Pr)_2NH$ (10 mL), 4 h, 85 ºC. Crude product was purified by flash column chromatography ($SiO_2$, toluene/ethyl acetate, 5:1 (v/v), 5% triethylamine) affording **4-C*p*** (0.39 g, 0.98 mmol, 87%) as a yellow solid. mp: 252.7–253.7 °C. $^1$H NMR (400 MHz, $CDCl_3$, δ): 8.63–8.62 (m, 4H), 7.73 (dd, *J* = 7.8, 0.7 Hz, 2H), 7.64 (dd, *J* = 1.5, 0.7 Hz, 2H), 7.56 (dd, *J* = 7.8, 1.5 Hz, 2H), 7.42–7.40 (m, 4H), 1.53 (s, 6H). $^{13}$C NMR (101 MHz, $CDCl_3$, δ): 154.24, 149.89, 139.53, 131.65, 131.43, 126.43, 125.65, 121.37, 120.70, 94.85, 87.37, 47.18, 27.03. HRMS–ASAP$^+$ (m/z): calcd for $C_{29}H_{20}N_2$ $[M]^{\bullet+}$, 396.1626; found, 396.1625. Anal. calcd for $C_{29}H_{20}N_2$: C 87.85; H 5.08; N 7.07; found: C 87.91; H 5.06; N 7.01.

**4,4'-bis(pyridin-4-ylethynyl)-1,1'-biphenyl (5-2H*p*)**

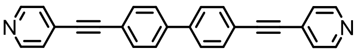

*General Procedure*: **24** (1.1 g, 6.4 mmol) in 1,4-dioxane (33 mL), TBAF (1M in THF, 6.4 mL), 30 min, rt. Cannulated into flask charged with dibromide **18** (0.50 g, 1.6 mmol), $PdCl_2(PhCN)_2$ (61 mg), CuI (18 mg), *t*-$Bu_3P$ (48 mg), and (*i*-$Pr)_2NH$ (15 mL), 2 h, 85 ºC. Crude product was purified by flash column chromatography ($SiO_2$, toluene/ethyl acetate, 5:1 (v/v), 5% triethylamine) affording **5-2H*p*** (0.48 g, 1.3 mmol, 85%) as a light yellow solid. mp: 250.0–250.9 °C. $^1$H NMR (400 MHz, $CDCl_3$, δ): 8.63–8.62 (m, 4H), 7.65 (s, 8H), 7.43–7.41 (m, 4H). $^{13}$C NMR (101 MHz, $CDCl_3$, δ): 149.76, 140.91, 132.61, 131.68, 127.22, 125.72, 121.69, 94.01, 87.83. HRMS–ASAP$^+$ (m/z): calcd for $C_{26}H_{17}N_2$ $[M+H]^+$, 357.1392; found, 357.1389. Anal. calcd for $C_{26}H_{16}N_2$: C 87.62; H 4.52; N 7.86; found: C 87.38; H 4.53; N 7.80.

## Synthesis of *meta*-Series

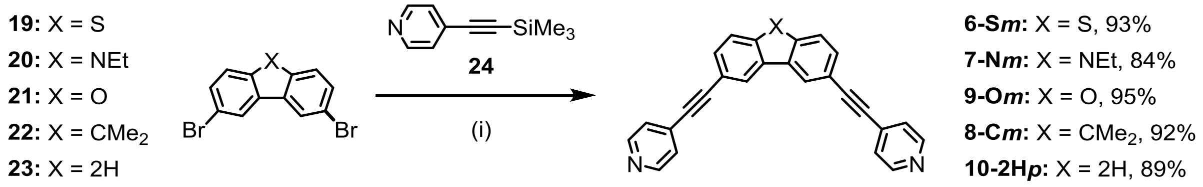


**Scheme 2.** Synthesis of *para* conjugated compounds **6–10**. Reagents and Conditions: (i) 4-((trimethylsilyl)ethynyl)pyridine **24**, TBAF (1 M in THF), 1,4-dioxane, rt, 30 min; then $PdCl_2(PhCN)_2$, CuI, *t*-$Bu_3P$, (*i*-Pr)$_2$NH, 85 °C, 3–12 h.

### 2,8-bis(pyridin-4-ylethynyl)dibenzo[b,d]thiophene (6-S*m*)

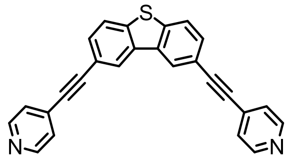


*General Procedure*: **24** (1.0 g, 5.7 mmol) in 1,4-dioxane (30 mL), TBAF (1 M in THF, 5.7 mL), 30 min, rt. Cannulated into flask charged with dibromide **19**[6] (0.50 g, 1.5 mmol), $PdCl_2(PhCN)_2$ (56 mg), CuI (17 mg), *t*-$Bu_3P$ (44 mg), and (*i*-Pr)$_2$NH (13 mL), 5 h, 85 ºC. Crude product was purified by flash column chromatography ($SiO_2$, toluene/ethyl acetate, 5:1 (v/v), 5% triethylamine) affording **6-S*m*** (0.52 g, 1.4 mmol, 93%) as a yellow solid. mp: 234.8–235.7 °C. $^1$H NMR (400 MHz, $CDCl_3$, δ): 8.65–8.63 (m, 4H), ), 8.38 (dd, *J* = 1.5, 0.6 Hz, 2H), 7.87 (d, *J* = 8.3, 0.6 Hz, 2H), 7.66 (dd, *J* = 8.3, 1.6 Hz, 2H), 7.44–7.43 (m, 4H). $^{13}$C NMR (101 MHz, $CDCl_3$, δ): 149.85, 140.89, 135.12, 131.62, 130.50, 125.68, 125.46, 123.20, 118.76, 94.14, 87.13. HRMS–ASAP$^+$ (m/z): calcd for $C_{26}H_{14}N_2S$ $[M]^{•+}$, 386.0878; found, 386.0876. Anal. calcd for $C_{26}H_{14}N_2S$: C 80.80; H 3.65; N 7.25; found: C 80.66; H 3.63; N 7.35.

**9-ethyl-3,6-bis(pyridin-4-ylethynyl)-9H-carbazole (7-N*m*)**

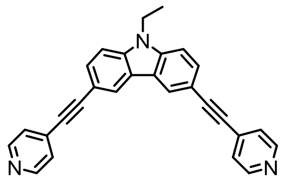

*General Procedure*: **24** (1.0 g, 5.7 mmol) in 1,4-dioxane (30 mL), TBAF (1 M in THF, 5.7 mL), 30 min, rt. Cannulated into flask charged with dibromide **20**[7] (0.50 g, 1.4 mmol), $PdCl_2(PhCN)_2$ (54 mg), CuI (16 mg), *t*-$Bu_3P$ (43 mg), and (*i*-Pr)$_2$NH (13 mL), 4 h, 85 ºC. Crude product was purified by flash column chromatography ($SiO_2$, toluene/ethyl acetate, 5:1 (v/v), 5% triethylamine) affording **7-N*m*** (0.47 g, 1.2 mmol, 84%) as a yellow solid. mp: 209.6–210.7 °C. $^1$H NMR (400 MHz, $CDCl_3$, δ): 8.62–8.60 (m, 4H), 8.31 (dd, *J* = 1.6, 0.6 Hz, 2H), 7.69 (dd, *J* = 8.4, 1.6 Hz, 2H), 7.43–7.41 (m, 6H), 4.39 (q, *J* = 7.2, 2H), 1.47 (t, *J* = 7.2, 3H). $^{13}$C NMR (101 MHz, $CDCl_3$, δ): 149.86, 140.62, 132.12, 130.28, 125.55, 124.90, 122.72, 113.05, 109.18, 95.60, 85.73, 38.13, 14.01. HRMS–ASAP$^+$ (m/z): calcd for $C_{28}H_{20}N_3$ $[M+H]^+$, 398.1657; found, 398.1639. Anal. calcd for $C_{28}H_{19}N_3$: C 84.61; H 4.82; N 10.57; found: C 84.56; H 4.74; N 10.51.

**2,8-bis(pyridin-4-ylethynyl)dibenzo[b,d]furan (8-O*m*)**

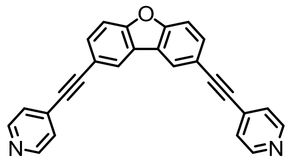

*General Procedure*: **24** (1.1 g, 6.1 mmol) in 1,4-dioxane (32 mL), TBAF (1 M in THF, 6.1 mL), 30 min, rt. Cannulated into flask charged with dibromide **21**[8] (0.50 g, 1.5 mmol), $PdCl_2(PhCN)_2$ (59 mg), CuI (18 mg), *t*-$Bu_3P$ (47 mg), and (*i*-Pr)$_2$NH (14 mL), 4 h, 85 ºC. Crude product was purified by flash column chromatography ($SiO_2$, toluene/ethyl acetate, 5:1 (v/v), 5% triethylamine) affording **8-O*m*** (0.54 g, 1.5 mmol, 95%) as a white solid. mp: 211.0–212.5 °C. $^1$H NMR (400 MHz, $CDCl_3$, δ): 8.64 (br s, 4H), 8.17 (dd, *J* = 1.7, 0.7 Hz, 2H), 7.70 (dd, *J* = 8.5, 1.7 Hz, 2H), 7.60 (dd, *J* = 8.5, 0.7 Hz, 2H), 7.45–7.44 (m, 4H). $^{13}$C NMR (101 MHz, $CDCl_3$, δ): 156.85, 149.85, 131.88, 131.68, 125.68, 124.82, 124.04, 117.33,

112.44, 94.07, 86.40. HRMS–ASAP$^+$ (m/z): calcd for $C_{26}H_{15}N_2O$ $[M+H]^+$, 371.1184; found, 371.1185. Anal. calcd for $C_{26}H_{15}N_2O$: C 84.31; H 3.81; N 7.56; found: C 84.28; H 3.78; N 7.58.

**4,4'-((9,9-dimethyl-9H-fluorene-3,6-diyl)bis(ethyne-2,1-diyl))dipyridine (9-C*m*)**

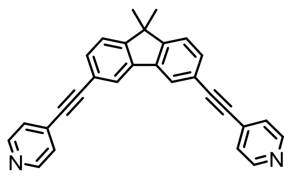


*General Procedure*: **24** (1.0 g, 5.7 mmol) in 1,4-dioxane (30 mL), TBAF (1 M in THF, 5.7 mL), 30 min, rt. Cannulated into flask charged with dibromide **22**[9] (0.50 g, 1.4 mmol), $PdCl_2(PhCN)_2$ (54 mg), CuI (16 mg), *t*-$Bu_3P$ (43 mg), and (*i*-Pr)$_2$NH (13 mL), 3 h, 85 ºC. Crude product was purified by flash column chromatography ($SiO_2$, toluene/ethyl acetate, 5:1 (v/v), 5% triethylamine) affording **9-C*m*** (0.51 g, 1.3 mmol, 92%) as a white solid. mp: 210.0–210.7 °C. $^1$H NMR (400 MHz, $CDCl_3$, δ): 8.64–8.62 (m, 4H), 7.93 (dd, $J$ = 1.5, 0.7 Hz, 2H), 7.55 (dd, $J$ = 7.8, 1.5 Hz, 2H), 7.46 (dd, $J$ = 7.8, 0.7 Hz, 2H), 7.44–7.42 (m, 4H), 1.52 (s, 6H). $^{13}$C NMR (101 MHz, $CDCl_3$, δ): 154.96, 149.66, 138.76, 131.97, 131.77, 125.78, 123.81, 123.09, 121.07, 94.70, 86.53, 47.54, 26.97. HRMS–ASAP$^+$ (m/z): calcd for $C_{29}H_{20}N_2$ $[M]^{\bullet+}$, 396.1626; found, 396.1625. Anal. calcd for $C_{29}H_{20}N_2$: C 87.85; H 5.08; N 7.07; found: C 88.08; H 5.02; N 7.13.

**3,3'-bis(pyridin-4-ylethynyl)-1,1'-biphenyl (10-2H*m*)**

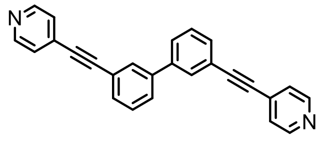


*General Procedure*: **24** (1.1 g, 6.1 mmol) in 1,4-dioxane (32 mL), TBAF (1 M in THF, 6.1 mL), 30 min, rt. Cannulated into flask charged with dibromide **23**[10] (0.50 g, 1.6 mmol), $PdCl_2(PhCN)_2$ (61 mg), CuI (18 mg), *t*-$Bu_3P$ (49 mg), and (*i*-Pr)$_2$NH (15 mL), 12 h, 85 ºC. Crude product was purified by flash column chromatography ($SiO_2$, toluene/ethyl acetate, 5:1

(v/v), 5% triethylamine) affording **10-2H*m*** (0.51 g, 1.4 mmol, 89%) as a white solid. mp: 173.2–174.5 °C. $^{1}$H NMR (400 MHz, $CDCl_3$, δ): 8.63–8.61 (m, 4H), ), 7.81 (td, *J* = 1.5, 0.6 Hz, 2H), 7.63 (dt, *J* = 7.7, 1.5 Hz, 2H), 7.57 (dt, *J* = 7.7, 1.5 Hz, 2H), 7.48 (td, *J* = 7.7, 0.6 Hz, 2H), 7.41–7.40 (m, 4H). $^{13}$C NMR (101 MHz, $CDCl_3$, δ): 149.87, 140.61, 131.48, 131.22, 130.67, 129.26, 128.10, 125.69, 122.88, 93.85, 87.12. HRMS–ASAP$^{+}$ (m/z): calcd for $C_{26}H_{17}N_2$ $[M+H]^{+}$, 357.1392; found, 357.1380. Anal. calcd for $C_{26}H_{16}N_2$: C 87.62; H 4.52; N 7.86; found: C 87.72; H 4.51; N 7.85.

**Supplementary Note 2. Absorption Spectra of Compounds 1–10.**

The UV–Vis absorption spectra of the compounds **1–10** were measured in a dilute and aerated dichloromethane solution at room temperature (Supplementary Figure 1). The optical band gaps of all the studied molecules were also calculated from the onset of optical absorption and are listed in Supplementary Table 1. To understand how the substitution pattern affects the optical energy band gap ($E_g$), we compare the *para*-linked structures **1–5** with the *meta*-linked structures **6–10**. Considering the effect of conjugation, compounds **1–5** show a smaller HOMO–LUMO gap, as the anchor group is attached in the *para* position. However, when the anchor group is attached at the *meta* position the $E_g$ is increased, supporting the hypothesis of reduced conjugation throughout the molecular wire. To observe any solvatochromic effects the UV–Vis absorption spectra of compounds **2** and **7** were recorded in various solvents (Supplementary Figure 2 and Supplementary Figure 3). *Para*-linked carbazole compound **2** shows no solvatochromism in the investigated solvents (ethanol, dichloromethane, and toluene). However, for the *meta*-linked carbazole **7** a marginal bathochromic shift (hypsochromic shift) of the longest wavelength transition was observed in ethanol (toluene) compared with the spectrum measured in dichloromethane. A comparison between theoretical calculated HOMO–LUMO gaps of the isolated molecules and their HOMO–LUMO gaps when anchored to gold clusters, alongside the experimental $E_g$ is shown in Supplementary Table 2. It is well known that DFT tends to underestimate HOMO–LUMO band gaps. For example for $C_{60}$, the HOMO-LUMO gap is 2.3 eV[11], whereas DFT predicts 1.2 – 1.5 eV[12,13]. On the other hand the relevant gap in a transport measurement is smaller than the gas phase gap and depends on the spacing between the electrodes[14].

**Supplementary Note 3. Molecular electronic properties.**

Detailed results of the molecular electronic properties measured by MCBJ are shown in Supplementary Figure 24 – Supplementary Figure 33.

**Supplementary Note 4. Computational Data. Binding energy of a set of molecules on gold surface.**

To calculate the optimum binding distance for a **4-C*p*** molecule between two gold (111) surfaces we used the SIESTA implementation of DFT and the counterpoise method, which removes basis set superposition errors (BSSE). The binding distance $z$ is defined as the distance between the gold surface and the molecule (**4-C*p***) at the closest point. **4-C*p*** is defined as monomer A and the gold electrodes as monomer B.

The ground state energy of the total system is calculated using SIESTA and is denoted $E_{AB}^{AB}$, with the parameters defined as those in the "Theory and Simulation" section of the main text. The gold leads consist of 3 layers containing 25 atoms. The energy of each monomer is then calculated in a fixed basis, which is achieved by the use of ghost atoms in SIESTA. Hence the energy of the individual molecule **4-C*p*** in the presence of the fixed basis is defined as $E_{A}^{AB}$ and for the gold is $E_{B}^{AB}$. The binding energy is then calculated using the following equation:

$$\text{Binding Energy} = E_{AB}^{AB} - E_{A}^{AB} - E_{B}^{AB} \qquad (1)$$

**Supplementary Figure 35**. shows the optimum binding distance $z$ is 2.3 Å.

**Supplementary Note 5. Tight binding calculation of Single Molecular *para*- and *meta*-linked Heterocycles**

To understand the role of pendant groups bridging the two phenyl rings, we now examine electron transport through a minimal tight-binding model of the structures shown in Supplementary Figure 44, in which the pendant moiety is represented by a single-site of orbital of energy $\varepsilon_0$ and all other site are assigned site energies of zero. The pendant site is connected to its nearest neighbours by a coupling matrix element $-\alpha$ and all other sites are connected to their nearest neighbours by a matrix element of magnitude unity. When $\alpha = 0$,

the pendant site is disconnected from the two six-membered rings and therefore the model reduces to that of a biphenyl molecule connected to external electrodes. Our aim is to examine the changes in electrical conductance, which arise when $\alpha$ changes from zero (pendant site disconnected) to unity (pendant site fully connected).

When identical one-dimensional external leads are connected to sites $i$ and $j$ of a scattering region (such as those shown in Supplementary Figure 44 and Supplementary Figure 45) by couplings $-\gamma_L$ and $-\gamma_R$ respectively, the transmission amplitude is[15]

$$t = 2isin(k)e^{2ik}\frac{\gamma_L\gamma_R}{\gamma}\frac{G_{ij}}{\Delta} \qquad (2)$$

In this equation, k (E) is the wavevector of the incident (and transmitted) electron of energy $E$,

$$\Delta = 1 + e^{ik}\left[G_{jj}\gamma_R^2 + G_{ii}\gamma_L^2\right] + \frac{d\, e^{2ik}\,\gamma_R^2\,\gamma_L^2}{\gamma^2} \qquad (3)$$

$$d = \left[G_{ii}G_{jj} - G_{ij}\,G_{ji}\right] \qquad (4)$$

and $G_{ij}$ is an element of the 13x13 Green's function matrix of the isolated core obtained when $\gamma_L = \gamma_R = 0$. Numerically, this is given by

$$G = (E - h)^{-1} \qquad (5),$$

where $h$ is the 13x13 hamiltonian of the isolated core. Analytically $G$ can be obtained by solving Dyson's equation as follows.

**Analytical formula for *G***

To obtain the core Green's function $G$ for the isolated core of Supplementary Figure 45, we first consider the case where $\alpha = \beta = 0$. In this case, apart from the isolated site $\varepsilon_b$, the

structure consists of two isolated 6-membered rings labelled A and B with Green's functions (equation (2)). As noted in ref[15], the Green's function of the 6-membered rings are given by

$$(g_A)_{ix} = C\cos(k(|i-x|-3))$$

$$(g_B)_{yj} = C\cos(k(|y-j|-3))$$

where $i, x$ are sites belonging to the left ring A and $j, y$ belong to the right ring B and

$$C = \frac{1}{2\gamma\sin(k)\sin(kN/2)}$$

When $\gamma_L$ and $\gamma_R$ are non-zero, it is convenient to view the above lattice as an example of the more general structure shown in Supplementary Figure 45, in which two subsystems labelled A and B are coupled to each other via sites $x, y$ by a single matrix element $-\beta$ and to a pendant site by $-\alpha$.

Then after solving Dyson's equation we obtain,

$$G_{ij} = (\hat{g}_{AB})_{ji} + \frac{\alpha^2}{E-\varepsilon_b-\Sigma}[(\hat{g}_{AA})_{i\,a} + (\hat{g}_{AB})_{i\,b}][(\hat{g}_{AB})_{aj} + (\hat{g}_{BB})_{\,bj}] \quad (6)$$

where

$$\Sigma = \alpha^2[(\hat{g}_{AA})_{aa} + (\hat{g}_{AB})_{ab} + (\hat{g}_{BA})_{ba} + (\hat{g}_{BB})_{bb}]$$

$$(\hat{g}_{AA})_{ia} = (g_A)_{ia} + \frac{\beta^2\,(g_A)_{ix}(g_B)_{yy}(g_A)_{xa}}{1-\,x_A}$$

$$(\hat{g}_{AB})_{ib} = \frac{-\beta\,(g_A)_{ix}(g_B)_{yb}}{1-\,x_A}$$

$$(\hat{g}_{AB})_{aj} = \frac{-\beta\,(g_A)_{ax}(g_B)_{yj}}{1-\,x_A}$$

$$(\hat{g}_{BB})_{bj} = (g_B)_{bj} + \frac{\beta^2\,(g_A)_{by}(g_B)_{xx}(g_A)_{ya}}{1-\,x_A}$$

$$(\hat{g}_{AB})_{ij} = \frac{-\beta\ (g_A)_{ix}(g_B)_{yj}}{1 - x_A}$$

$$x_A = (g_A)_{xx}(g_B)_{yy}\,\beta^2$$

An interesting prediction from equation (6) is that when $E = \varepsilon_b$ the transmission coefficient is independent of $\alpha$. For $\beta = 1$ and $\varepsilon_b = 0$ Supplementary Figure 46 shows examples of $T$ (E) versus $E$ for different values of $\alpha$ when $\varepsilon_b = 0$, while Supplementary Figure 47 shows the corresponding results when $\varepsilon_b = 0.5$. As expected, these curves coincide when $E = \varepsilon_b$.

## Supplementary References


1. Clayden, J., Greeves, N. & Warren, S. *Organic Chemistry*. (OUP Oxford, 2012).
2. Dias, F. B. *et al.* Dipolar Stabilization of Emissive Singlet Charge Transfer Excited States in Polyfluorene Copolymers. *J. Phys. Chem. B* **112,** 6557–6566 (2008).
3. Liu, X. *et al.* A 2,7-carbazole-based dicationic salt for fluorescence detection of nucleic acids and two-photon fluorescence imaging of RNA in nucleoli and cytoplasm. *Org. Biomol. Chem.* **9,** 3615–3618 (2011).
4. Wang, S. *et al.* Dicationic dibenzofuran derivatives as anti-Pneumocystis carinii pneumonia agents: synthesis, DNA binding affinity, and anti-P. carinii activity in an immunosuppressed rat model. *Eur. J. Med. Chem.* **34,** 215–224 (1999).
5. Singh, V., Wang, S. & Kool, E. T. Genetically Encoded Multispectral Labeling of Proteins with Polyfluorophores on a DNA Backbone. *J. Am. Chem. Soc.* **135,** 6184–6191 (2013).
6. Yang, W. *et al.* Improvement of color purity in blue-emitting polyfluorene by copolymerization with dibenzothiophene. *J. Mater. Chem.* **13,** 1351–1355 (2003).

7. Esra Oğuztürk, H., Tirkeş, S. & Önal, A. M. Electrochemical synthesis of new conjugated polymers based on carbazole and furan units. *J. Electroanal. Chem.* **750,** 1–8 (2015).
8. Zhang, S. *et al.* Tuning the Optoelectronic Properties of 4,4′-N,N′-Dicarbazole-biphenyl through Heteroatom Linkage: New Host Materials for Phosphorescent Organic Light-Emitting Diodes. *Org. Lett.* **12,** 3438–3441 (2010).
9. Kobin, B., Grubert, L., Blumstengel, S., Henneberger, F. & Hecht, S. Vacuum-processable ladder-type oligophenylenes for organic–inorganic hybrid structures: synthesis, optical and electrochemical properties upon increasing planarization as well as thin film growth. *J. Mater. Chem.* **22,** 4383–4390 (2012).
10. Maji, M. & Studer, A. Transition-Metal-Free Oxidative Homocoupling of Aryl, Alkenyl, and Alkynyl Grignard Reagents with TEMPO. *Synthesis* **2009,** 2467–2470 (2009).
11. Lof, R. W., van Veenendaal, M. A., Koopmans, B., Jonkman, H. T. & Sawatzky, G. A. Band gap, excitons, and Coulomb interaction in solid $C_{60}$. *Phys. Rev. Lett.* **68,** 3924–3927 (1992).
12. Saito, S. & Oshiyama, A. Cohesive mechanism and energy bands of solid $C_{60}$. *Phys. Rev. Lett.* **66,** 2637–2640 (1991).
13. Troullier, N. & Martins, J. L. Structural and electronic properties of $C_{60}$. *Phys. Rev. B* **46,** 1754–1765 (1992).
14. Perrin, M. L. *et al.* Large tunable image-charge effects in single-molecule junctions. *Nat. Nanotechnol.* **8,** 282–287 (2013).
15. Lambert, C. J. Basic concepts of quantum interference and electron transport in single-molecule electronics. *Chem. Soc. Rev.* **44,** 875–888 (2015).
